# Development and Evaluation of a Web-Based App for Adverse Effect Management in Breast Cancer Patients Treated with Oral Targeted Therapy or Chemotherapy: Findings from a Pilot Study

**DOI:** 10.3390/curroncol33050272

**Published:** 2026-05-07

**Authors:** Julie Lemieux, Isabelle Côté, Martine Lemay, Sophie Lauzier, Philippe Després, Christine Desbiens, Catherine Doyle, Brigitte Poirier, Amel Baghdadli, Leonardo Di Schiavi Trotta, Hermann Nabi

**Affiliations:** 1 Centre des Maladies du Sein, CHU de Québec—Université Laval, Quebec City, QC G1S 4L8, Canada; isabelle.cote@chudequebec.ca (I.C.); martine.lemay@chudequebec.ca (M.L.); christine.desbiens.med@ssss.gouv.qc.ca (C.D.); catherine.doyle.med@ssss.gouv.qc.ca (C.D.); brigitte.poirier.med@ssss.gouv.qc.ca (B.P.); 2 Centre de Recherche du CHU de Québec—Université Laval, Quebec City, QC G1V 4G2, Canada; sophie.lauzier@crchudequebec.ulaval.ca (S.L.); philippe.despres@phy.ulaval.ca (P.D.); amel.baghdadli@crchudequebec.ulaval.ca (A.B.); leonardo.di-schiavi-trotta@crchudequebec.ulaval.ca (L.D.S.T.); hermann.nabi@crchudequebec.ulaval.ca (H.N.)

**Keywords:** breast cancer, chemotherapy, administration, oral, drug-related side effects and adverse reactions, mobile applications

## Abstract

This pilot study (NCT05743686) evaluated the feasibility of a web-based application enabling patients with breast cancer (BC) receiving oral therapy to self-report and manage treatment-related adverse effects (AEs). Patients were enrolled between January and August 2023. Historical controls were used for comparison. Participants used the web-app to self-report AEs daily for 13 weeks, completed the PRO-CTCAE weekly, and completed a questionnaire assessing psychosocial precursor factors associated with treatment adherence. Some patients and healthcare professionals participated in semi-structured interviews. The study included 28 participants and 185 historical controls. Compared with controls, participants had fewer interactions with hospital pharmacists (0 [0–1] vs. 0 [0–7], *p* = 0.04), with no significant differences in the number of visits, hospitalizations, or modifications to treatment. Concordance between AEs reported via the web-app and PRO-CTCAE was 66.2%. No statistically significant changes were observed in psychosocial precursor factors of treatment adherence following the intervention (all *p* > 0.05). The qualitative data underscored the generally positive reception of the web-based application among both patients and healthcare professionals. In conclusion, in this small mixed-methods pilot study, monitoring oral cancer therapy-related adverse effects using the web-app was feasible and acceptable, and was associated with a lower frequency of telephone contacts with hospital pharmacists compared with historical usual care. These preliminary findings are exploratory and warrant confirmation in larger, prospectively designed studies.

## Simple Summary

This pilot study evaluated a web-based application developed to support patients with breast cancer receiving oral anticancer therapy in monitoring, managing, and reporting treatment-related side effects from home. Over a 13-week period, participants used the application to record daily symptoms and complete brief questionnaires regarding their well-being and treatment experiences. The findings indicate that both patients and healthcare professionals perceived the application as acceptable and feasible. Its use was associated with a reduction in telephone interactions with hospital pharmacists, suggesting a potential decrease in healthcare workload. Although no significant improvements were observed in treatment adherence-related measures, these preliminary results support the application’s value as a supportive tool for patient self-management and communication. The insights gained from this pilot study may inform future research and guide the integration of digital health technologies into routine oncology care.

## 1. Introduction

Breast cancer is the most frequently diagnosed cancer in women worldwide and remains a major cause of morbidity and mortality [1,2]. Over time, the treatment paradigm for hormone receptor (HR)-positive, HER2-negative metastatic breast cancer has evolved considerably. The introduction of oral CDK4/6 inhibitors has expanded therapeutic options for patients with HR-positive, HER2-negative disease [3]. Additional oral systemic treatments include mTOR inhibitors [4], capecitabine [5], and newer targeted agents such as alpelisib [6], inavolisib [7], and capivasertib [8]. Several of these agents have also received approval for use in the adjuvant setting [5,9,10].

Although oral agents provide greater convenience than hospital-administered intravenous chemotherapy, their at-home use introduces distinct challenges, particularly in terms of monitoring and managing adverse effects and ensuring treatment adherence [11]. Whereas intravenous chemotherapy requires regular hospital visits, allowing close monitoring by oncologists, pharmacists, nurses, and other healthcare professionals, home-based oral therapies shift the responsibility for treatment management largely to patients. Despite their convenience, these oral agents may still cause clinically significant adverse effects, potentially leading to poor adherence, suboptimal treatment outcomes, reduced quality of life, and increased healthcare utilization and costs [12,13]. Therefore, developing accessible and convenient strategies to monitor and manage adverse effects associated with oral therapies is essential.

In the current Information Age, most individuals have Internet access and routinely use smartphones, tablets, or computers. Patient-reported outcome measure (PROM) systems that allow patients to self-report treatment-related adverse effects have been shown to improve survival among individuals with cancer [14] and to reduce healthcare costs [15]. Furthermore, these digital tools can promote patient engagement in disease management, enhancing autonomy and empowerment [16,17].

Accordingly, this pilot study aimed to develop and evaluate the feasibility and implementation of a web-based application (web-app) designed to help patients with breast cancer receiving oral therapy self-report and manage treatment-related adverse effects. The specific objectives were to (1) adapt existing information on oral therapeutic agents for integration into a mobile web-based platform; (2) develop algorithms to support adverse effect management and follow-up scheduling; and (3) design interactive content linking patient-reported side effects with tailored, web-app-generated recommendations. The study also sought to assess the web-app’s performance in a real-world clinical setting, including its impact on healthcare team workload relative to standard care, patient and healthcare professional satisfaction with the digital tool, and the scalability and transferability of the management module to hospitals across the Province of Quebec. Changes in dose modification, temporary treatment interruption, and discontinuation were also explored. This study was designed as a pilot mixed-methods feasibility evaluation, with the primary aim of assessing the acceptability and practical implementation of the web-app, rather than testing definitive hypotheses regarding clinical outcomes.

## 2. Materials and Methods

### 2.1. Study Design and Participants

This mixed-methods pilot prospective study enrolled patients with breast cancer receiving oral systemic therapy at the Centre des maladies du sein, CHU de Québec—Université Laval, between January and August 2023. The study protocol was approved by the Research Ethics Committee of the CHU de Québec—Université Laval (approval number: 2021-5644, 17 September 2021) and was registered at ClinicalTrials.gov (NCT05743686). The study was conducted in accordance with the principles of the Declaration of Helsinki and its subsequent amendments, as well as Good Clinical Practice guidelines. Written informed consent was obtained from all participants for the prospective component of the study.

Eligible patients met the following criteria: (1) a confirmed diagnosis of breast cancer; (2) initiation of treatment with palbociclib, ribociclib, abemaciclib, capecitabine, or everolimus; (3) ownership of a smartphone, tablet, or computer with Internet access and the ability to use these devices; (4) fluency in spoken and written French; and (5) age ≥ 18 years.

The target sample size (approximately 30 participants) was chosen pragmatically to allow evaluation of feasibility, adherence to the intervention, and variability of key outcomes over the recruitment period, rather than based on a formal power calculation for between-group comparisons.

### 2.2. Design and Development of the Treatment-Management Algorithms and Web-App

A hospital pharmacist developed treatment-management algorithms addressing the principal adverse effects associated with each oral therapeutic agent used in breast cancer, based on management recommendations from the Groupe d’Étude en Oncologie du Québec (https://www.geoq.info/) and the respective product monographs. These algorithms were subsequently reviewed and validated by a panel of five oncology pharmacists from the Centre des maladies du sein, CHU de Québec—Université Laval. The final versions of the algorithms are provided in File S1.

A computer specialist then implemented the algorithms using REDCap (Research Electronic Data Capture) [18,19], hosted on secure servers at the CHU de Québec—Université Laval Research Center (https://projectredcap.org/resources/citations/ (accessed on 24 February 2026)). At the time of the study, no institutional patient portal was available at the CHU de Québec—Université Laval; therefore, REDCap was used as the patient-facing web interface for this project.

### 2.3. Pilot Intervention

Upon enrollment, the participant’s information and therapy type were entered into REDCap. Starting on the first day of treatment, the participant received an email at 4:00 a.m. each day, asking whether they were experiencing side effects. The participant could follow the link to answer “yes” or “no”. If the patient answered yes, they were then asked questions about the nature and severity of the adverse effects. Participants who reported grade I or II adverse effects received on-screen guidance with basic self-management instructions, whereas those reporting grade III adverse effects were automatically directed to seek immediate care at the nearest emergency department, and the on-duty pharmacist received an email requesting that they contact the participant as soon as possible. All self-reported adverse effects were documented in the patient’s chart.

Every 7 days, participants were asked to complete the French version of the PRO-CTCAE form, tailored to the most common symptoms associated with their specific medication, online. The weekly PRO-CTCAE form served to determine the concordance with the daily symptom reports submitted during the preceding week. Each participant remained in the study for up to 13 weeks, or for a shorter period if their oral therapy was discontinued. A participant who answered “no” was considered to have experienced no adverse effect on that day. For the primary analysis of daily app-based symptom reports, calendar days without a completed entry were coded as “no adverse event reported” to permit uniform aggregation of day-level data over the observation period. This approach assumed that non-completion was more likely to reflect absence of clinically significant symptoms or non-engagement with the app rather than unreported toxicity; however, this assumption may not hold for all participants and could lead to underestimation of adverse events in the web-app cohort. In a sensitivity analysis, days with missing app entries were excluded from agreement analyses between daily web-app reports and weekly PRO-CTCAE, rather than being coded as “no adverse event.”

Every week, the adverse effect report was sent to the electronic patient chart.

### 2.4. Control Group

To assess the outcomes associated with the use of the web-app compared with usual care, a retrospective cohort was established. Patients who were prescribed palbociclib, ribociclib, abemaciclib, capecitabine, or everolimus between 1 September and 31 December 2023 were identified through the electronic patient chart system. Data for this cohort were extracted for a period of 13 weeks following initiation of oral therapy.

### 2.5. Outcomes

The study outcomes were as follows: (1) workload of healthcare professionals, assessed based on the number of telephone calls made by patients to, or received from, pharmacists and nurse navigators, the number of unplanned consultations (by telephone or in person) with oncologists, the number of emergency department visits, and the number of hospitalizations; (2) concordance between adverse effects reported via the web-app and those captured using validated patient-reported outcome measures for adverse events (i.e., the PRO-CTCAE questionnaires); (3) psychosocial precursor factors of treatment adherence; and (4) treatment modifications, including dose adjustments, temporary interruptions, or discontinuations.

The number of telephone and in-person consultations and treatment modifications were extracted from patient medical records for both participants and controls by an independent registrar who was not otherwise involved in the study. Unplanned consultations with oncologists were identified by comparing actual visits with those scheduled in the preceding clinical plan. For example, if the oncologist’s note stated, “to be seen in 3 months,” but the patient was seen after 1 or 2 months, the visit was classified as unplanned; conversely, if the patient was seen at 3 months or later, the visit was classified as planned.

In the intervention group, concordance between daily web-app reports and weekly PRO-CTCAE data was examined. As there is currently no summary scoring system for the PRO-CTCAE (https://healthcaredelivery.cancer.gov/pro-ctcae/faqs.html#scored (accessed on 10 June 2024)), each item response in the present study was assigned a numeric value from 0 to 4 (0 = never/not at all; 1 = light/a little; 2 = moderate/moderately; 3 = severe/a lot; 4 = very severe/constantly). Weekly PRO-CTCAE scores were obtained by summing the numeric responses across items. For the web-app, weekly scores were calculated as the sum of all reported events during the week multiplied by their reported grade.

Concordance was defined as both tools (web-app and PRO-CTCAE) indicating either no adverse effects or the presence of adverse effects (i.e., scores = 0 for both, or ≥1 for both). Discordance occurred when one tool reported adverse effects (score ≥ 1) while the other did not (score = 0); in such cases, the instrument reporting no adverse effects was considered discordant. Because the two tools used distinct scoring methods, scores were not directly comparable.

Psychosocial precursor factors of adherence (intention, attitude, subjective norm, perceived behavioral control, social support, coping planning, and anticipated regret) were assessed using previously developed Likert-7 scales [20].

For both the control and intervention groups, data on telephone and in-person consultations and treatment modifications were obtained from patient medical records by an independent registrar who was not involved in other aspects of the study. No patient-reported outcome measures (PROMs) were available for the control group.

### 2.6. Additional Variables Collected

Demographic variables (sex, age, ethnicity, education, employment status, marital status, income, and time spent on the Internet per week), cancer characteristics (histological type, histological grade, stage, estrogen receptor, progesterone receptor, and HER2 status), and treatment-related information (type of neoadjuvant and/or adjuvant chemotherapy, type of adjuvant hormonal therapy, radiotherapy, and line of metastatic treatment, if applicable) were extracted from patient medical records.

For the historical control cohort, additional potentially relevant baseline and clinical variables, such as performance status, ethnicity, and patient-reported measures, were not systematically recorded in a standardized format during the historical period and therefore could not be reliably abstracted for all patients.

### 2.7. Statistical Analysis

Given the pilot nature of the study and the small intervention sample, all between-group comparisons and *p*-values should be interpreted as descriptive and hypothesis-generating. The study was not powered to detect statistically significant differences between cohorts or across individual agents.

All statistical analyses were performed using R, version 4.5.2 (The R Project for Statistical Computing). Continuous variables were assessed for normal distribution using the Shapiro–Wilk test. Normally distributed variables were presented as means ± standard deviations and analyzed using Student’s *t*-test. Non-normally distributed variables were presented as medians (range) and analyzed using the Mann–Whitney U-test. Categorical variables were presented as n (%) and compared using the chi-squared test or Fisher’s exact test, as appropriate.

Associations between adverse effect scores obtained from the web-app and the PRO-CTCAE were examined using Spearman’s rank correlation, and agreement between the two tools was quantified using Cohen’s κ; these are preliminary measures of agreement designed to assess whether the app captures broadly similar patterns to PRO-CTCAE. Two-sided *p*-values < 0.05 were considered statistically significant.

### 2.8. Qualitative Component of the Study

A subsample of patients (n = 7) and healthcare professionals (n = 5) participated in semi-structured interviews. Patients were purposively selected to capture variation in age, cancer stage, and type of targeted or oral chemotherapy, whereas healthcare professionals were chosen to represent diverse medical backgrounds, including oncologists, pharmacists, and nurse navigators. Telephone interviews were conducted by team members trained in social and cultural anthropology and/or psychosocial oncology (SL, Julie Lapointe) (File S2). Interview transcripts were analyzed thematically by a research professional with expertise in qualitative methodology (AB) using a mixed inductive–deductive codebook approach and NVivo (v12) [21,22,23]. All participant quotations were translated into English by a professional translator.

## 3. Results

### 3.1. Characteristics of the Participants and Historical Controls

Between January and August 2023, the study was presented to 38 patients, of whom 28 agreed and provided informed consent to participate. The characteristics of the participants are summarized in Table 1. The study also included a historical control cohort comprising 185 patients treated between 1 September and 31 December 2023 (Table 2). There were no significant differences in age between the two cohorts (*p* = 0.24).

### 3.2. Feasibility Metrics

The daily completion rate of web-app symptom reporting varied substantially among participants, ranging from 10% (in one participant who completed entries almost exclusively when experiencing an adverse effect) to 96%. The median completion rate was 69%. Between 22 and 27 participants completed the PRO-CTCAE each week. There were no true dropouts; although all participants missed at least one week of PRO-CTCAE completion, these omissions occurred inconsistently across individuals. Data on healthcare workload are presented in the following section.

### 3.3. Calls/Visits to Healthcare Professionals

Participants in the web-app group made fewer telephone consultations with hospital pharmacists compared with controls (0.07 events per participant; median 0 [range, 0–1] vs. 0.4 events per patient; median 0 [range, 0–7]; *p* = 0.04). No significant differences were observed between groups for the other resource utilization measures (Table 3).

### 3.4. Treatment Characteristics, Adverse Effect Reporting, and Adherence Factors

There were no statistically significant differences between the intervention and control groups in treatment measures (all *p* > 0.05) (Table 3). In the web-app group, all treatment modifications (dose adjustment, n = 3; temporary interruption, n = 2; discontinuation, n = 2) were attributable to adverse effects related to capecitabine (n = 5) or abemaciclib (n = 2). In the control group, treatment changes (dose adjustment, n = 32; temporary interruption, n = 29; discontinuation, n = 23) were clearly linked to adverse effects in 77.4% of cases; however, due to information bias arising from inconsistencies in adverse effect reporting among healthcare professionals, formal comparisons between groups were not feasible, and these findings should be interpreted as exploratory. In the control group, 12 treatment modifications occurred in patients receiving abemaciclib, 20 in those receiving ribociclib, 23 in those receiving capecitabine, 3 in those receiving everolimus, and 26 in those receiving palbociclib.

In the intervention group, overall concordance between the web-app and the PRO-CTCAE was 66.2%, with the web-app responsible for discordance in 85.3% of instances (i.e., the web-app reporting no adverse effects while the PRO-CTCAE score was >1) (Table 4). In a sensitivity analysis (Table A1), days with missing app entries were excluded from agreement analyses between daily web-app reports and weekly PRO-CTCAE, rather than coded as “no adverse event.” In that situation, agreement between daily web-app reports and weekly PRO-CTCAE increased to 72.0% (vs. 66.2% in the primary analysis), with the web-app responsible for discordance in 82.4% of cases (vs. 85.3% in the primary analysis) (Appendix A), suggesting that assumptions regarding missing data exerted a moderate influence on agreement estimates.

The overall Cohen’s κ was 0.40. For diarrhea, concordance was 74.8% with a Cohen’s κ of 0.45; for nausea, concordance was 79.4% with a Cohen’s κ of 0.49; for stomatitis, concordance was 95.1% with a Cohen’s κ of 0.28; and for hand–foot syndrome, concordance was 91.2% with a Cohen’s κ of 0.63. Nonetheless, web-app and PRO-CTCAE scores were moderately correlated (r = 0.50, *p* < 0.0001) (Table 5). There were no significant differences in psychosocial precursor factors of treatment adherence between baseline and post-intervention assessments (all *p* > 0.05) (Table 6).

### 3.5. Interview Findings

#### 3.5.1. Patient Interviews

Seven metastatic patients (39–75 years) reported generally high usability and acceptability of the web-app. It was perceived as simple, clear, and largely non-intrusive, with most participants engaging regularly despite variable adherence. Daily prompts were acceptable overall, though some found them repetitive, especially when asymptomatic. The tool fostered a strong sense of support and reassurance, particularly through daily monitoring and rapid care-team follow-up. It was valued as a communication channel and for reinforcing symptom management information. However, some participants experienced burden, describing the app as repetitive or as a constant reminder of illness. For a minority, it did not meaningfully alter their care experience. Participants highlighted a lack of personalization and humanization, noting rigidity and limited warmth in interactions. Suggested improvements included tailoring message frequency to clinical status, adding options for “no symptoms,” enabling more flexible and detailed symptom reporting, incorporating dynamic/adaptive questionnaires, and increasing direct human contact (e.g., call requests, personalized messages). Detailed quotes are provided in File S3.

#### 3.5.2. Healthcare Professional Interviews

Healthcare professionals reported good acceptability, noting that the web-app supported clinical practice by structuring patient discussions, enabling targeted interventions, and functioning as a screening tool that helped patients report and reflect on symptoms. It encouraged more proactive management and increased patient–team interactions, though some clinicians experienced uncertainty and added workload due to alerts prompting follow-up decisions. For others, it did not substantially change follow-up practices. Implementation was broadly supported. Key facilitators included integration with existing systems, multidisciplinary involvement, clear guidance and training, technical support, and patient partner engagement. Barriers included time burden for onboarding and support, limited clarity around processes, suboptimal information sharing, and difficulty identifying active users or available reports. Concerns were also raised about usability for older adults, those with low digital literacy, and cognitively impaired patients. Detailed quotes are provided in File S4.

## 4. Discussion

This pilot prospective study evaluated the feasibility of a web-app for self-reporting and managing treatment-related adverse effects in patients with breast cancer receiving oral therapies. The findings support feasibility and acceptability in routine practice, with reduced healthcare team workload and no clear impact on healthcare utilization (dose adjustments, interruptions, discontinuations) or psychosocial precursors of adherence. Qualitative data from patients and healthcare professionals indicated overall positive experiences. Given the small sample (n = 28), heterogeneous treatments, and use of a non-concurrent historical control, results should be considered preliminary and hypothesis-generating.

Digital tools are increasingly used to support adherence, toxicity monitoring, and patient–provider communication in breast cancer, particularly for oral targeted therapies (e.g., CDK4/6 and PI3K/mTOR inhibitors) [24]. Multicomponent interventions show modest and heterogeneous adherence benefits, with greater effects than reminder-only strategies [24,25,26,27]. Non-adherence remains common (>40% in some cohorts) and clinically consequential [28]. Given the toxicity profiles of these agents, structured monitoring tools may facilitate early detection and management [29,30].

Comparison of daily web-app reports with weekly PRO-CTCAE [31] showed that adverse effects were more often captured in the weekly instrument, despite its susceptibility to recall bias [32]. Interviews suggested incomplete daily reporting when patients were asymptomatic and confusion about the role of the weekly questionnaire. Discrepancies likely reflect single daily entries (limiting capture of evolving symptoms), recall effects, differing perceptions of clinical consequences, and reporting burden (daily completion 10–96% vs. weekly 79–96%). These findings highlight the need for clear communication about the tool purpose and optimized reporting design.

Healthcare utilization analyses suggested fewer pharmacist telephone contacts among web-app users versus controls, though this exploratory finding requires confirmation due to small sample size and potential confounding. Qualitative data indicate that perceived monitoring and timely feedback may reduce unnecessary contacts while supporting early toxicity management. Prior evidence shows that ePRO-based monitoring can improve outcomes and reduce costs in oncology [15,20,33,34,35].

No significant differences were observed in dose modification, interruption, or discontinuation, and adherence was not robustly measured (chart-based data only), limiting inference [12,13]. Although digital interventions can improve adherence [36], the short follow-up, limited intensity, and sample size may explain null findings for adherence-related outcomes.

Qualitative findings emphasized high usability, perceived support, and improved communication, alongside variable engagement and preferences for reminder frequency [26,37,38]. Key limitations included limited personalization, rigid questionnaires, and ambiguity between clinical monitoring and research use. Healthcare professionals reported benefits for structuring discussions and enabling proactive care, but noted uncertainty around alert management and minimal impact on workflow for some [37,38,39]. Implementation facilitators included integration, training, and multidisciplinary involvement, while barriers included onboarding time, identification of active users, and challenges for patients with low digital literacy or cognitive impairment [40,41,42,43].

Participants proposed refinements to enhance flexibility and humanization: adaptive reporting frequency, a “no symptoms” option, expanded and open-text symptom reporting, clearer guidance on severity, within-day updates, and integrated options to request clinician contact. These changes may improve usability, data completeness, and alignment with patient needs.

Scale-up beyond the study site was not achieved due to administrative and regulatory barriers for clinical software approval and the emergence of similar compliant commercial solutions.

This study has several important limitations. As a small pilot without a formal power calculation, it was not designed to provide definitive comparative effectiveness estimates, and the limited sample size constrains precision and generalizability. In addition, substantial heterogeneity in oral therapies (e.g., CDK4/6 inhibitors, capecitabine, everolimus), each with distinct toxicity profiles and monitoring requirements, complicates the interpretation of toxicity and utilization outcomes. The pragmatic use of a retrospectively collected historical control group further limits comparability, with incomplete baseline characterization (e.g., ethnicity) and imbalance in disease setting (mixed adjuvant/metastatic in the intervention group vs. exclusively metastatic controls) and treatment distribution, introducing potential confounding. Taken together, the small sample size, treatment heterogeneity, and non-concurrent historical control design mean that all effect estimates should be interpreted with caution and regarded as exploratory. Additional data gaps, including a lack of information on community pharmacist contacts, visits to other centers (restricted by confidentiality regulations), and the magnitude of dose adjustments, further limit inference. Confirmation in larger, adequately powered, prospective multicenter studies with standardized data collection and contemporaneous comparison groups is required.

Importantly, missing daily web-app reports were treated as “no adverse event,” which may have led to under-reporting if non-response was related to symptom severity. Although sensitivity analyses suggested only moderate effects on agreement with weekly PRO-CTCAE, findings should be interpreted cautiously.

Heterogeneity of oral therapies further limits interpretation. CDK4/6 inhibitors, capecitabine, and everolimus have distinct toxicity profiles and monitoring requirements [44,45,46]. Pooling these regimens complicates toxicity comparisons and supports an exploratory framing. Future studies should use more homogeneous cohorts or enable regimen-specific analyses.

Finally, emerging molecular heterogeneity (e.g., microRNA and signaling pathway variation) may influence toxicity and treatment tolerance [47,48]. As therapies become more individualized, flexible, patient-centered digital monitoring tools may help capture diverse toxicity patterns and generate real-world data for integration with translational research.

## 5. Conclusions

Use of a REDCap-based web-app for patient self-reporting of adverse effects during oral therapy in breast cancer is feasible and acceptable in real-world oncology practice. Although not designed for definitive effectiveness, this pilot provides initial signals of reduced workload for hospital pharmacists and highlights patterns of agreement and discrepancy with PRO-CTCAE reporting. Mixed-methods findings further identify practical design and implementation considerations, including usability, personalization, alert management, and workflow integration. These results are preliminary and hypothesis-generating. Larger, multicenter, adequately powered prospective studies are required to confirm effectiveness, clarify impacts on clinical outcomes and resource utilization, and optimize implementation strategies across diverse oncology settings.

## Figures and Tables

**Table 1:** Characteristics of the participants.

Characteristics	Values
n	28
Age, years, median (range)	49.4 (36.4–74.7)
Ethnicity, n (%)	
Caucasian	25 (89.3)
Black	2 (7.1)
Latin-American	1 (3.6)
Education, n (%)	
High school	2 (7.1)
Vocational school	13 (46.4)
University certificate	4 (14.3)
Bachelor’s degree	8 (28.6)
Graduate studies	1 (3.6)
Marital status, n (%)	
Single	6 (21.4)
Single parent, with children at home	2 (7.1)
Married, without children at home	9 (32.1)
Married, with children at home	11 (39.3)
Annual personal income, Canadian dollars/year, n (%)	
<10,000	1 (3.6)
20,000–29,999	3 (10.7)
30,000–39,999	1 (3.6)
40,000–49,999	1 (3.6)
50,000–59,999	4 (14.3)
60,000–79,999	3 (10.7)
80,000–99,999	3 (10.7)
100,000–119,999	4 (14.3)
≥120,000	5 (17.9)
Prefer not to disclose	3 (10.7)
Main electronic tool, n (%)	
Computer	9 (32.1)
Tablet	5 (17.9)
Smartphone	14 (50.0)
Weekly time spent on the Internet, h, median (range)	8 (1–50)
Histological subtype, n (%)	
Ductal	25 (89.3)
Lobular	2 (7.1)
Mucinous	1 (3.6)
Histological grade, n (%)	
1	0
2	18 (64.3)
3	10 (35.7)
Staging at initial presentation, n (%)	
I	7 (25.0)
II	11 (32.3)
III	2 (7.1)
IV	8 (28.6)
Receptors, positive, n (%)	
Estrogen receptors	25 (89.3)
Progesterone receptors	19 (67.9)
HER2	1 (3.6)
Adjuvant treatments, n (%)	
Chemotherapy	15 (53.6)
Hormonal therapy	13 (46.4)
Radiotherapy	19 (67.9)
Treatment line when entering the study, n (%)	
Adjuvant	3 (10.7)
First metastatic	15 (53.6)
Second metastatic	5 (17.9)
Third metastatic	3 (10.7)
Fourth metastatic	2 (7.1)

**Table 2:** Characteristics of the historical controls compared with the participants.

Characteristics	Web-App Participants	Historical Controls
n	28	185
Age, years, median (range)	49.4 (36.4–74.7)	52.6 (35.1–79.1)
Treatment setting, n (%)		
Adjuvant	3 (10.7)	0
Metastatic	25 (89.3)	185 (100)
Treatment, n (%)		
Abemaciclib	9 (32.1)	15 (8.1)
Ribociclib	8 (28.6)	43 (23.2)
Capecitabine	9 (32.1)	37 (20.0)
Palbociclib	1 (3.6)	90 (48.7)
Everolimus	1 (3.6)	0

**Table 3:** Secondary outcomes.

Characteristics	Web-App Participants (n = 28)				Historical Controls (n = 185)				*p*
	Event/n	Median	IQR	Range	Event/n	Median	IQR	Range	
Number of calls/visits, per patient, over 13 weeks after starting treatment									
Hospital pharmacists, telephonic consultations	0.07	0	0–0	0–1	0.4	0	0–0	0–7	0.04
Nurse navigators, telephonic consultations	1.4	1	0–3	0–6	1.3	1	0–2	0–14	0.55
Oncologists, consultations (telephonic and visits)	2.1	2	1–3	0–5	2.6	2	1–3	1–7	0.25
Emergency room visits	0.1	0	0–0	0–1	0.2	0	0–0	0–4	0.92
Hospitalizations	0.04	0	0–0	0–1	0.05	0	0–0	0–1	0.86
	n (%)				n (%)				*p*
Modifications in treatment									
Dose adjustment	3 (10.7)				32 (17.3)				0.58
Temporary interruption	2 (7.1)				29 (15.7)				0.39
Discontinuation	2 (7.1)				23 (12.4)				0.75

The number of calls/visits are shown as median (IQR; range).

**Table 4:** Concordance in self-reported adverse effects between the App and PRO-CTCAE.

Week	PRO-CTCAE			Web-App			Concordance? n/N (%)	Non-Concordance	
	Median	IQR	Range	Median	IQR	Range		Due to PRO-CTCAE, n/N (%) *	Due to Web-App, n/N (%) ^#^
1	2	1–3.25	0–13	2	0–4	0–17	18/27 (63.0)	1/9 (11.1)	8/9 (88.9)
2	3	1–5	0–8	2	0–8	0–29	20/27 (74.1)	0	7/7 (100)
3	2	1–3.75	0–6	2	0–5	0–14	18/26 (69.2)	2/8 (25.0)	6/8 (75.0)
4	2	1–3	0–6	1	0–4.5	0–22	16/25 (64.0)	2/9 (22.2)	7/9 (78.8)
5	2	0.25–3	0–11	2	0–7.5	0–21	19/25 (76.0)	1/6 (16.7)	5/6 (83.3)
6	2	0–4	0–7	0	0–6	0–15	16/23 (69.6)	0	7/7 (100)
7	2	1–4	0–7	1	0–4	0–14	18/26 (69.2)	0	8/8 (100)
8	3	1–5	0–6	2.5	0–6.75	0–23	17/24 (70.8)	1/7 (14.3)	6/7 (85.7)
9	2	0–2	0–5	0	0–5	0–21	13/25 (52.0)	3/12 (25.0)	9/12 (75.0)
10	2	1–3	0–6	2	0–6.5	0–14	17/25 (68.0)	0	8/8 (100)
11	2	1–3.5	0–12	0	0–4.25	0–14	14/26 (53.9)	1/12 (8.3)	11/12 (91.7)
12	2	1–3	0–5	2	0–6	0–11	12/22 (54.6)	4/10 (40.0)	6/10 (60.0)
13	1	1–2	0–6	2	0–2.75	0–7	18/24 (75.0)	1/6 (16.7)	5/6 (83.3)
Overall	2	1–3	0–13	2	0–5	0–29	216/325 (66.2)	16/109 (14.7)	93/109 (85.3)

Calendar days without a completed web-app entry were coded as “no adverse event reported.” * PRO-CTCAE reported no events, while events were reported using the web-app. ^#^ The web-app reported no events, while events were reported using the PRO-CTCAE.

**Table 5:** Spearman correlations between the PRO-CTCAE and web-app scores.

Week	r	*p*
1	0.462	0.018
2	0.537	0.004
3	0.668	0.0004
4	0.608	0.002
5	0.582	0.003
6	0.393	0.064
7	0.362	0.070
8	0.582	0.004
9	0.272	0.209
10	0.314	0.127
11	0.587	0.002
12	0.432	0.073
13	0.432	0.038
Overall	0.500	<0.0001

**Table 6:** Changes in psychosocial precursor factors of treatment adherence.

Dimension	Baseline			End of Study			*p*_pre-post_
	Median	IQR	Range	Median	IQR	Range	
Intention	7	7–7	6–7	7	7–7	1–7	0.27
Perceived behavioral control	6.8	6.25–7	3.2–7	6.6	6.15–7	2–7	0.60
Subjective norm	7	6–7	5–7	7	6.5–7	5.3–7	0.83
Anticipated regret	6.3	5.1–7	3.2–7	6.4	5.55–7	1.8–7	0.91
Perception of social support—Relatives	7	6.67–7	4.67–7	7	5.75–7	1.33–7	0.12
Perception of social support—Health professionals	6.67	6.08–7	4.33–7	6.83	6–7	1–7	0.63
Attitude	1.5	1–2.44	1–7	1.38	1–2	1–3	0.19
Coping planning—Side effects	6.33	6–7	5–7	6.33	6–7	5–7	0.15
Coping planning—Routine	6.83	6–7	5–7	7	6.67–7	6–7	0.18

All scores are on 7.

## Data Availability

Anonymized datasets can be made available upon reasonable request to the Corresponding Author.

## Supplementary Materials

The following supporting information can be downloaded at https://www.mdpi.com/article/10.3390/curroncol33050272/s1. File S1. Algorithms; File S2. Interview guides; File S3. Results of semi-structured interviews with women; File S4. Results of semi-structured interviews with health professionals.

## Abbreviations

The following abbreviations are used in this manuscript:

AE	Adverse event
HR	Hormone receptor
MEMS	Medication Event Monitoring System
PROM	Patient-reported measure
Web-app	Web-based application

## Appendix A

**Table A1:** Sensitivity analysis of the concordance in self-reported adverse effects between the app and PRO-CTCAE.

Week	PRO-CTCAE			Web-App			Concordance? n/N (%)	Non-Concordance	
	Median	IQR	Range	Median	IQR	Range		Due to PRO-CTCAE, n/N (%) *	Due to Web-App, n/N (%) ^#^
1	2	1–3.25	0–13	2	0–4	0–17	18/27 (63.0)	1/9 (11.1)	8/9 (88.9)
2	3	1–5	0–8	2	0–8	0–29	21/27 (77.8)	0	6/6 (100)
3	2	1–3.75	0–6	2	0–5	0–14	18/26 (69.2)	2/8 (25.0)	6/8 (75.0)
4	2	1–3	0–6	1	0–4.5	0–22	18/25 (72.0)	2/7 (28.6)	5/7 (31.4)
5	2	0.25–3	0.11	2	0–7.5	0–21	19/25 (76.0)	1/6 (16.7)	5/6 (83.3)
6	2	0–4	0–7	0	0–6	0–15	17/23 (73.9)	0	6/6 (100)
7	2	1–4	0–7	1	0–4	0–14	19/26 (73.1)	0	7/7 (100)
8	3	1–5	0–6	2.5	0–6.75	0–23	18/24 (75.0)	1/6 (16.7)	5/6 (83.3)
9	2	0–2	0–5	0	0–5	0–21	16/25 (64.0)	3/9 (33.3)	6/9 (66.7)
10	2	1–3	0–6	2	0–6.5	0–14	19/25 (76.0)	0	6/6 (100)
11	2	1–3.5	0–12	0	0–4.25	0–14	17/26 (65.4)	1/9 (11.1)	8/9 (88.9)
12	2	1–3	0–5	2	0–6	0–11	14/22 (63.6)	4/8 (50.0)	4/8 (50.0)
13	1	1–2	0–6	2	0–2.75	0–7	20/24 (83.3)	1/4 (25.0)	3/4 (75.0)
Overall	2	1–3	0–13	2	0–5	0–29	234/325 (72.0)	16/91 (17.6)	75/91 (82.4)

Calendar days without a completed web-app entry were excluded from the agreement analysis. * PRO-CTCAE reported no events, while events were reported using the web-app. ^#^ The web-app reported no events, while events were reported using the PRO-CTCAE.
