# Supplementary material for: Development and Evaluation of a Web-Based App for Adverse Effect Management in Breast Cancer Patients Treated with Oral Targeted Therapy or Chemotherapy: Findings from a Pilot Study
Source: Curr Oncol. 2026 May 7;33(5):272. doi: 10.3390/curroncol33050272 (PMC13205787; doi:10.3390/curroncol33050272)

SUPPLEMENTARY MATERIALS

ALGORITHMS

Abémaciclib- DIARRHÉES

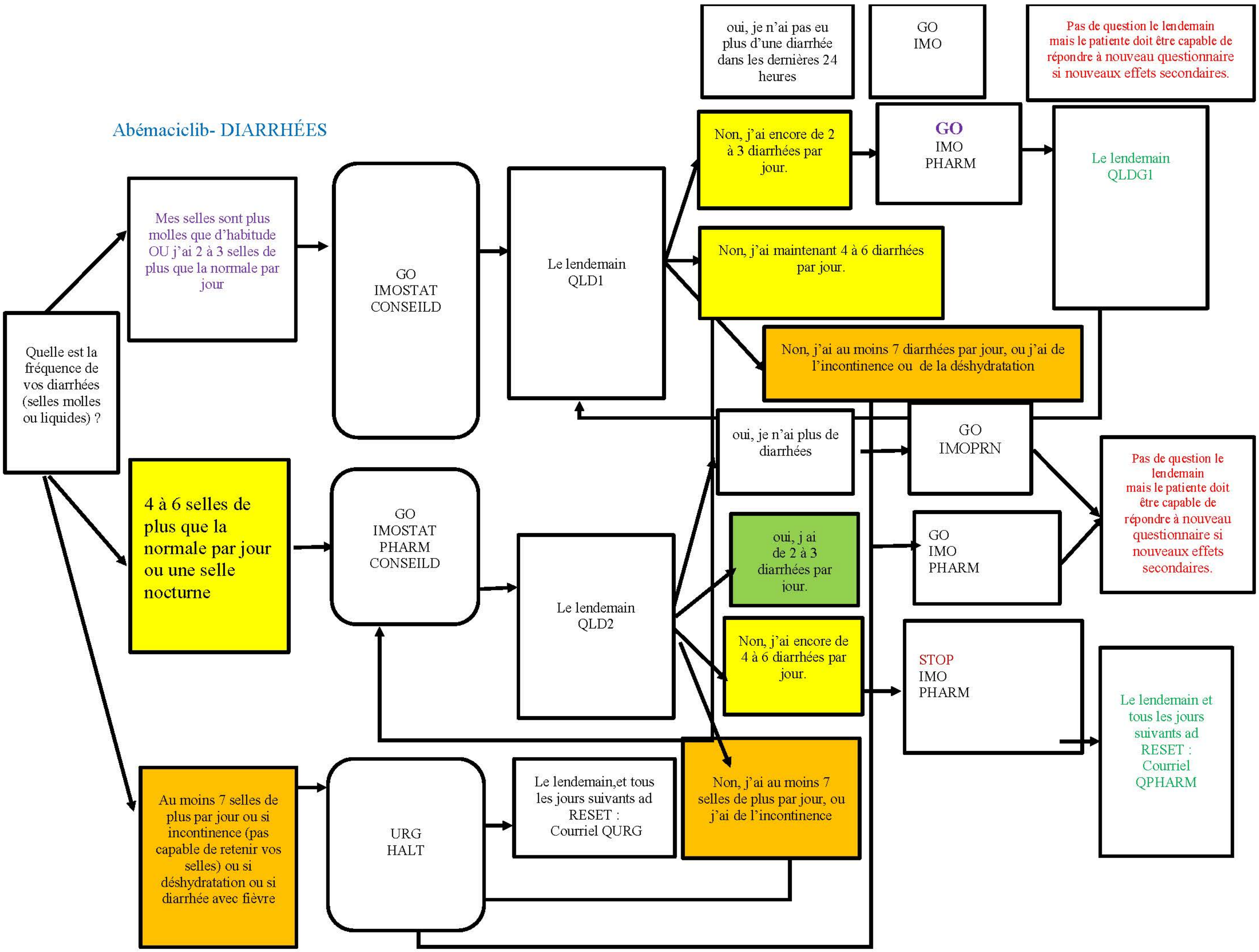

Palbociclib, ribociclib et évérolimus- DIARRHÉES

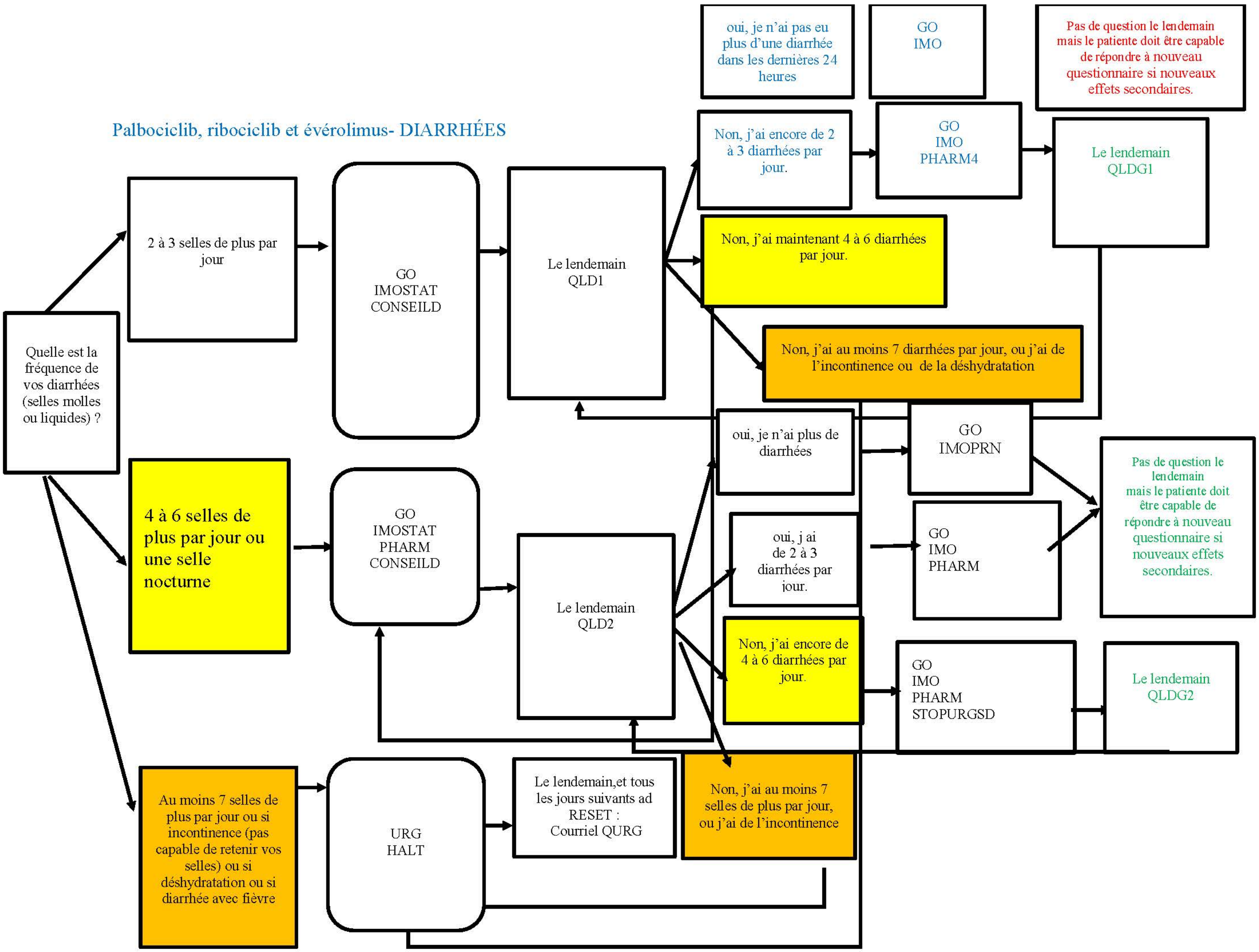

Palbociclib, ribociclib, abémaciclib, évérolimus Nausées et vomissements

Jaune et orange = pop-up pharmacie, rouge= changement par rapport à capécitabine

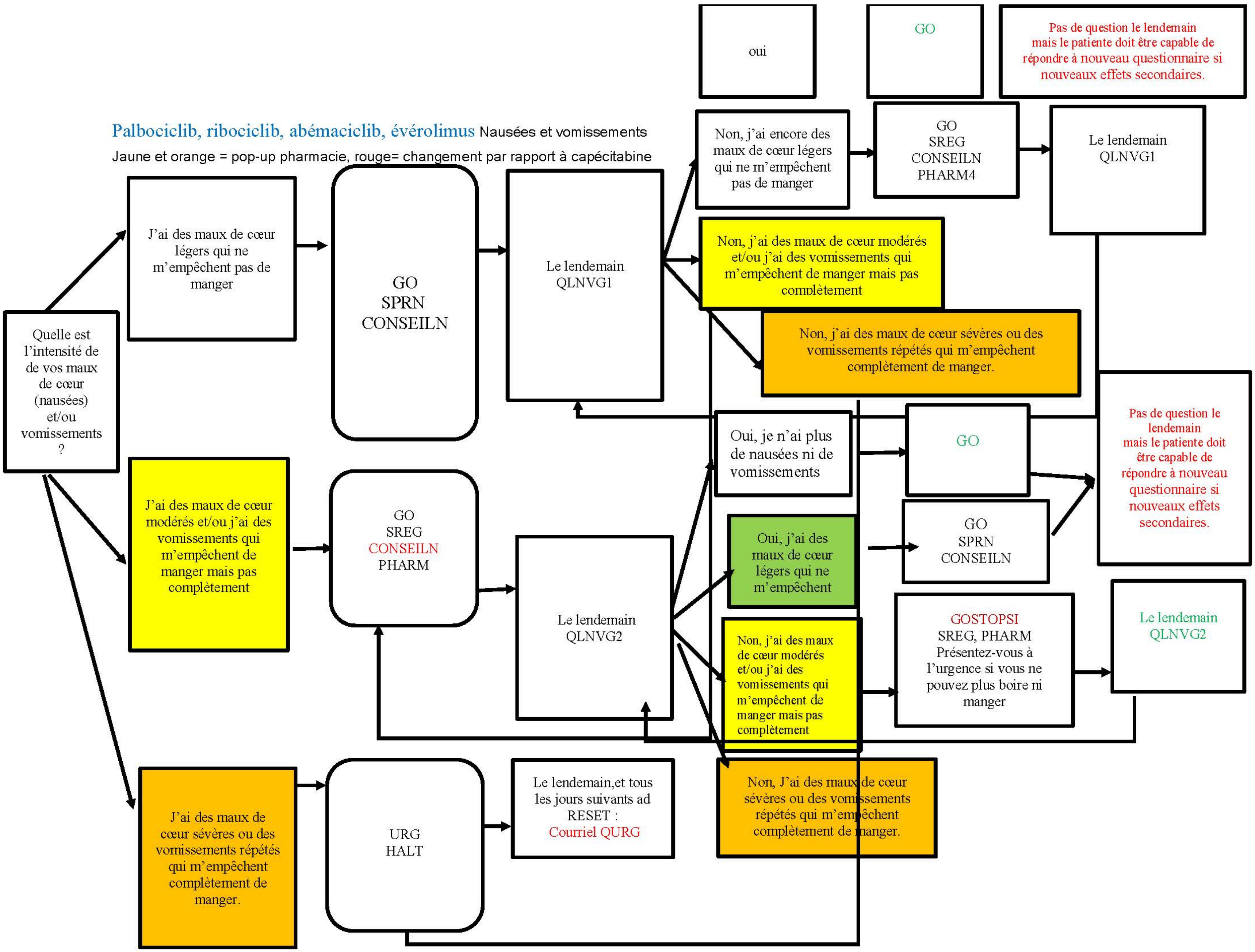

RASH- Ribociclib, palbociclib et évérolimus

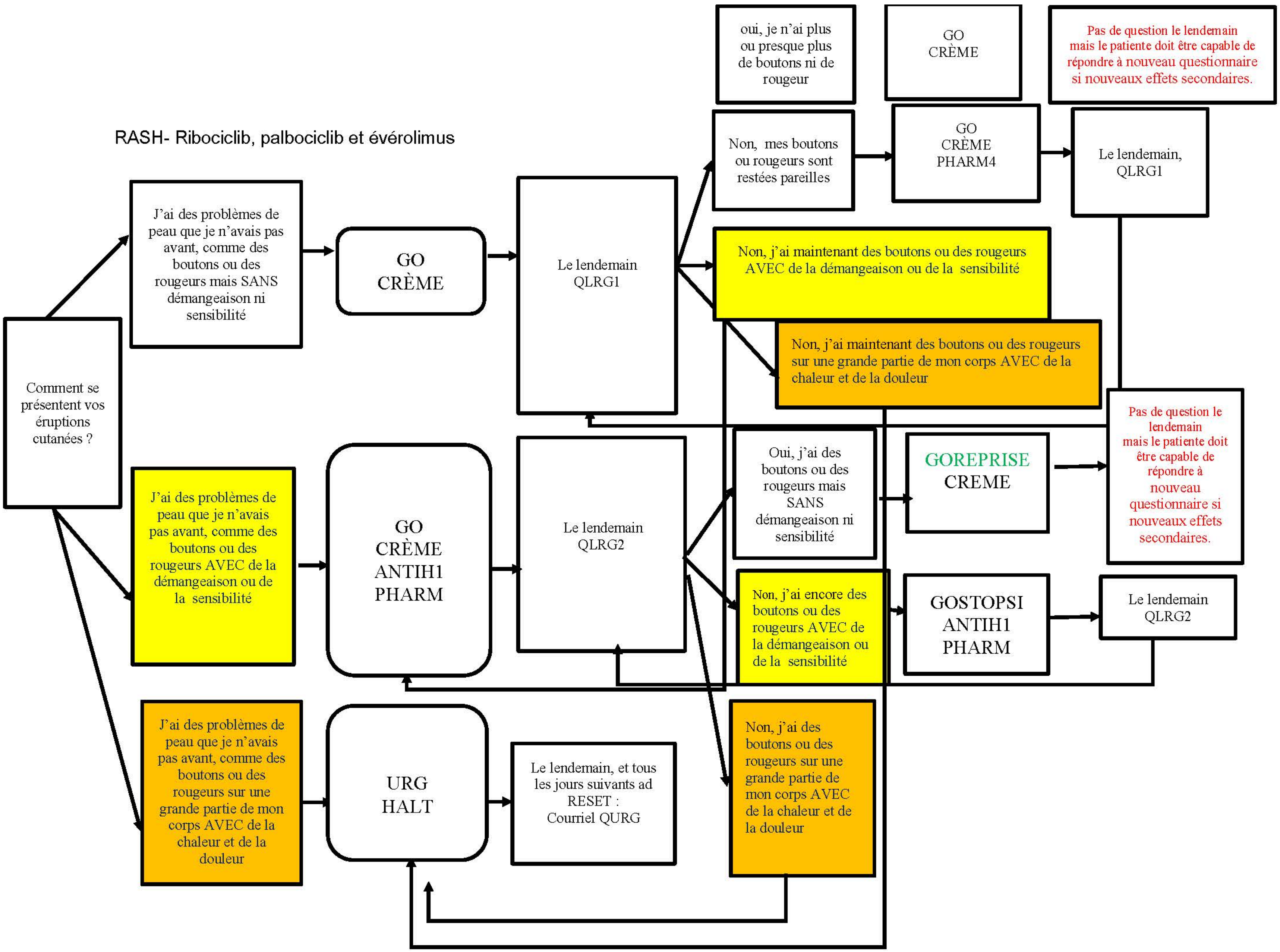

Évérolimus- Stomatite

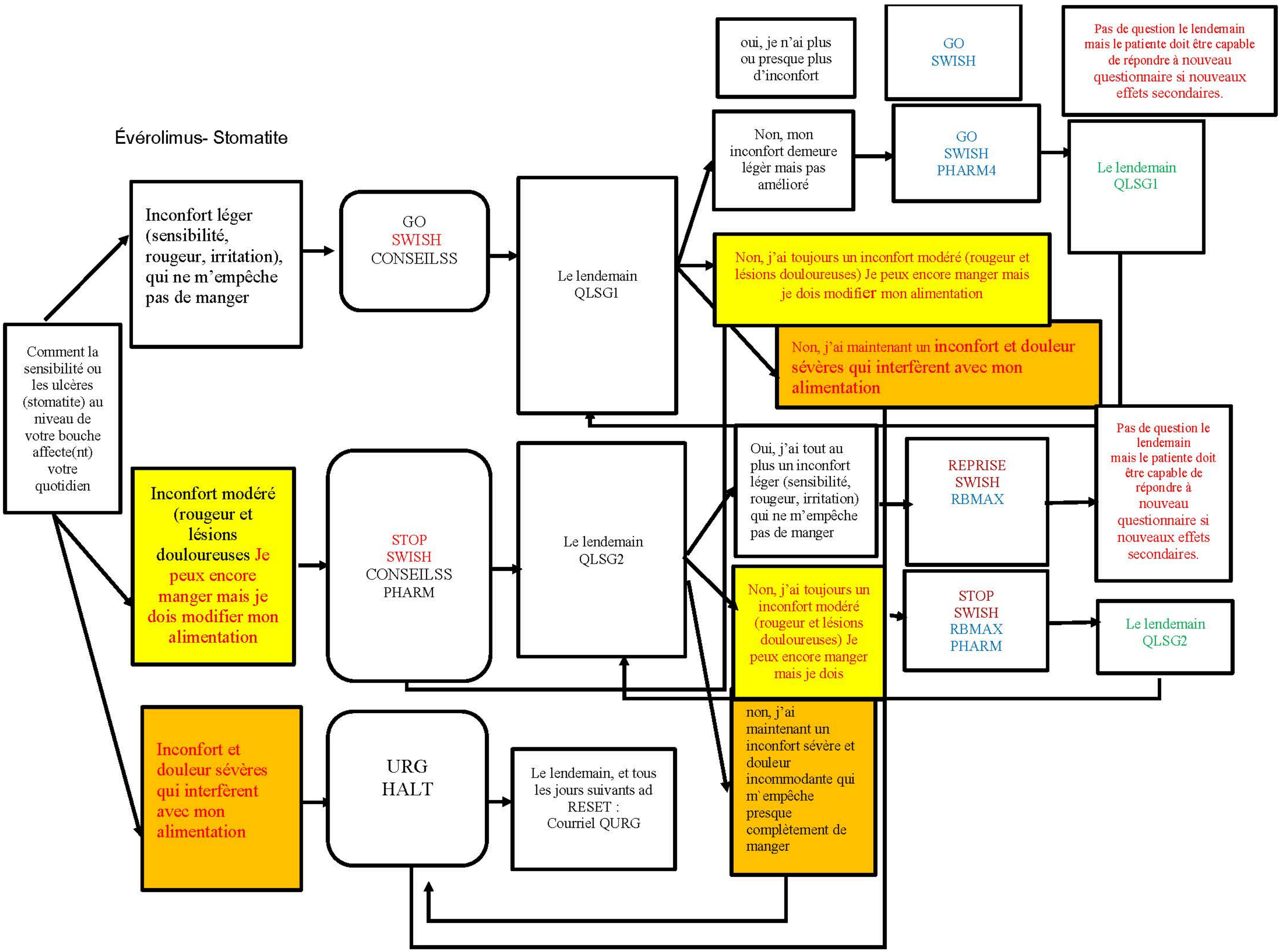

Palbociclib et ribociclib- Stomatite

Jaune = pop-up pharmacie,

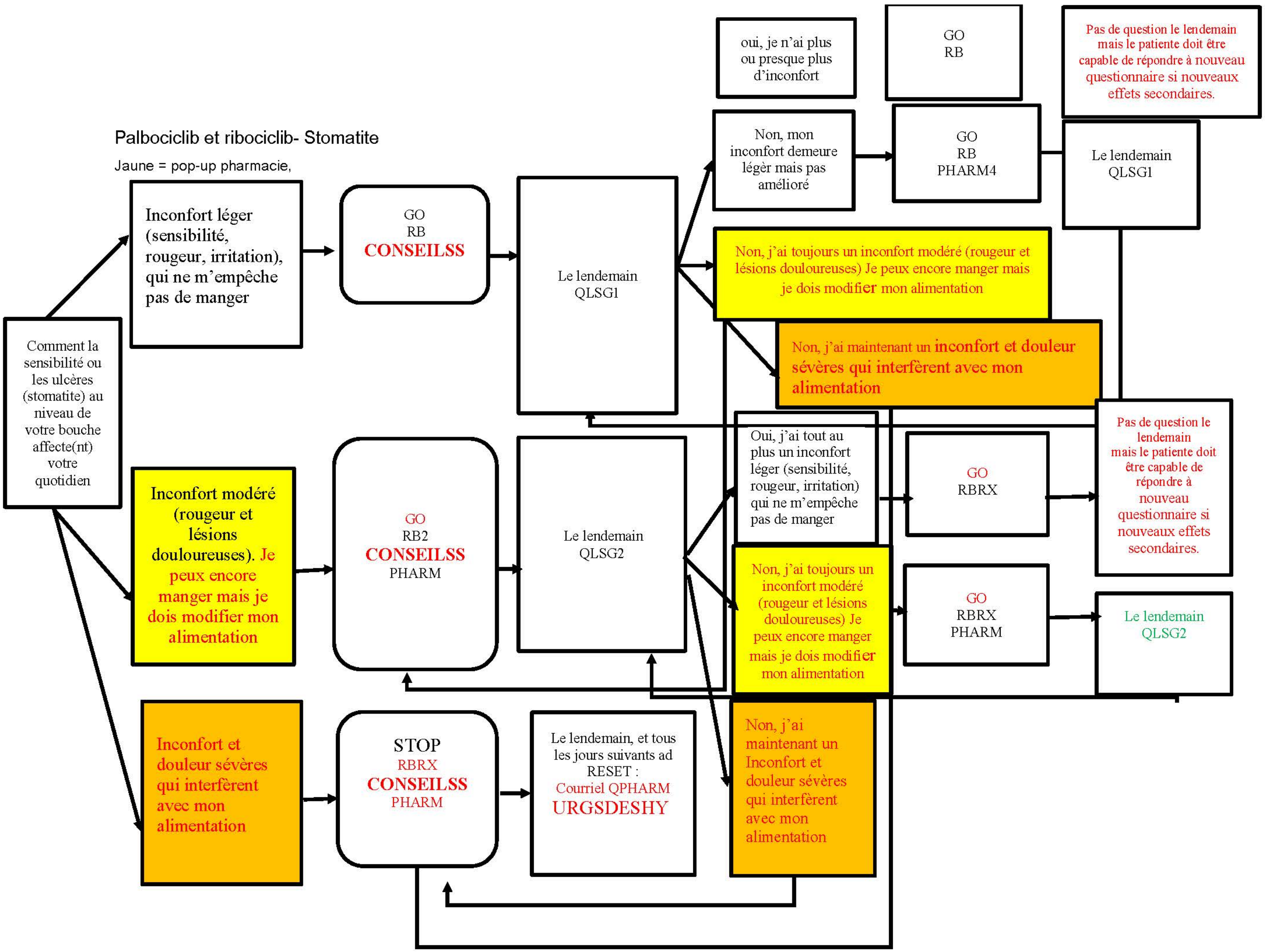

Supplement: Supplementary file 1 [file curroncol-33-00272-s001.zip › File S1 JCH.pdf]
