# Supplementary material for: Development and Evaluation of a Web-Based App for Adverse Effect Management in Breast Cancer Patients Treated with Oral Targeted Therapy or Chemotherapy: Findings from a Pilot Study
Source: Curr Oncol. 2026 May 7;33(5):272. doi: 10.3390/curroncol33050272 (PMC13205787; doi:10.3390/curroncol33050272)
Supplement: Supplementary file 1 [file curroncol-33-00272-s001.zip › File S2 JCH.pdf]

SUPPLEMENTARY MATERIALS

INTERVIEW GUIDES (ORIGINAL FRENCH)

GUIDE D'ENTREVUE INDIVIDUELLE POUR LES PATIENTS(TES)
Projet Appli

RENSEIGNEMENTS À COMPLÉTER PAR L'INTERVIEWEUR

Nom de l'intervieweur : ________________________________

Date de l'entrevue : ___________________________________

Modalité de l'entrevue (encercler) : Téléphone Teams

Durée de l'entrevue : __________________________________

Profil de la participante :

Stade de cancer : ________
Année du diagnostic :______________
Traitement concerné par l'étude : _________________

SECTION A – INTRODUCTION ET CONTEXTE

- Se présenter
- Demander l'autorisation d'enregistrer l'appel
- Expliquer le déroulement de l'entrevue :
    - Dans le cadre de l'étude à laquelle vous avez participé, il vous a été proposé de rapporter vos effets secondaires à l'aide d'une application internet.
    - Nous aimerions connaître votre expérience et votre appréciation de cette application.
    - Je vais vous poser des questions sur cette application et sur les soins et services que vous avez reçus.
    - Tout au long de l'entrevue, il est important de se rappeler qu'il n'y a pas de bonnes ou de mauvaise réponse.
    - Il est à noter également que je n'ai pas participé au développement de cet outil et que je ne suis pas impliquée dans l'équipe médicale.
    - Nous allons interviewer plusieurs femmes qui ont participé à cette étude. Lorsque les résultats seront présentés dans des rapports, des articles ou des conférences, ils seront rendu anonymes et il ne sera pas possible de vous identifier.
    - Ce que nous souhaitons avec cette entrevue c'est vraiment d'obtenir votre point de vue pour pouvoir ensuite améliorer cet outil.

Avant de débuter, j'aimerais savoir :

- À quel moment avez-vous reçu un diagnostic de cancer du sein?
- Pour quel traitement vous a-t-on suggéré d’utiliser cette application?

SECTION B – UTILISAITON DE L’APPLICATION

J’aimerais maintenant savoir comment s’est passé pour vous l’utilisation de cette application.

- À quel moment vous a-t-on proposé d’utiliser cette application?

- Parlez-moi de la manière dont vous avez utilisé l’application, en commençant par le tout début.
  - Au besoin :
    - Vous l’avez utilisé dès qu’il vous a été recommandé ou vous avez attendu un certain temps?
    - À quelle fréquence avez-vous utilisé l’application?
    - L’avez-vous utilisée pour rapporter des effets indésirables? Lesquels? Des exemples concrets.
    - Pour connaitre les recommandations? Ont-elles été appliquées? Des exemples concrets.

- Dans quelle mesure ce que vous avez communiqué avec cette application a conduit à des communications avec l’équipe médicale, qu’elles aient été initiées par vous ou un professionnel de la santé.
    - Qui? Comment? Pourquoi? Des exemples concrets.

- En plus d’utiliser l’application, est-ce que vous avez communiqué avec des professionnels de la santé pour discuter de vos effets indésirables?

SECTION C – APPRÉCIATION GLOBALE DE L’APPLICATION

Je vais maintenant vous poser des questions sur votre expérience avec l’ensemble des activités de l’application.

- Que pensez-vous des activités et des outils qui vous ont été offerts dans le cadre de l’application?
  - Au besoin :
    - Envoi par courriel?
    - Fréquence?
    - Évaluation des effets indésirables?
    - Conseils et recommandations?
    - Le fait que ce soit communiqué à l’équipe médicale?
    - La rétroaction des pharmaciens? De l’équipe médicale?
    - Les feuillets d’information sur le médicament?
    - Autre?

- Parlez-moi de ce que vous avez apprécié de l’application? Pour quelle(s) raisons?

- Parlez-moi de ce que vous avez moins apprécié de l’application? Pour quelle(s) raisons?

- À quel point il était pour vous facile ou difficile d’utiliser cette application?
  - Est-ce que vous avez eu besoin de l’aide d’une autre personne (proche, équipe médicale)?
  - Est-ce que des facteurs ont facilité/contraint l’utilisation de l’application?
  - Qu’est-ce qui aurait pu être fait pour faciliter votre utilisation?

- À quel point l'application a répondu à des besoins que vous aviez en lien avec votre traitement?
    - Lesquel(s) besoin(s) en particulier?
    - Pourriez-vous m'expliquer ce qui a fait que l'application a répondu/n'a pas répondu à vos besoins?
    - Est-ce qu'une composante de l'application a particulièrement permis de répondre à votre/vos besoins? Pour quelle(s) raison(s)?

- Croyez-vous que l'application a pu faire ou non une différence sur votre expérience avec votre traitement contre le cancer en comprimé?
    - Pouvez-vous m'expliquer ce qui vous amène à dire cela?
    - Quels impacts positifs/négatifs de l'application avez-vous perçus?
    - Est-ce qu'un aspect de l'application a eu un plus impact plus important que les autres? Si oui, laquelle et pour quelle(s) raison(s)?

- Est-ce que l'application vous a apporté certaines inquiétudes?

- Qu'est-ce qui devrait être fait pour améliorer une telle application?
- Recommanderiez-vous l'application à d'autres femmes ayant reçu une prescription de traitement contre le cancer en comprimé à la suite à un cancer du sein? Pour quelle(s) raison(s)?

SECTION D – APPRÉCIATION DES AUTRES SOINS ET SERVICES

- En plus de l'application, avez-vous reçu d'autres soins et services en lien avec votre traitement contre le cancer en comprimé, que ce soit à l'hôpital du Saint-Sacrement ou ailleurs? Si oui, lesquels?

- Quels soins et services avez-vous trouvez les plus/les moins utiles? Pour quelle(s) raison(s)?

- Recommanderiez-vous l'ajout de l'application aux soins et services qui sont habituellement offerts? Pour quelle(s) raison(s)?
    - Quel apport percevez-vous de l'application par rapport aux autres soins et services?

SECTION E – CONCLUSION

- Avez-vous d'autres idées de ce qui pourrait être fait pour mieux accompagner et outiller les femmes qui reçoivent une prescription d'un traitement contre le cancer en comprimé suite à un cancer du sein?

- Y-a-t-il d'autres éléments que vous aimeriez apporter concernant votre expérience avec le traitement contre le cancer en, les soins et services que vous avez reçus en lien avec ce médicament ou l'application?

Je vous remercie beaucoup pour le temps que vous m'avez accordé aujourd'hui.

SI LA PARTICIPANTE A DES QUESTIONS MÉDICALES OU MANIFESTE DE LA DÉTRESSE :

- Est-ce que vous acceptez que je fasse part de vos questions / votre situation à la personne qui coordonne l'étude au Centre des maladies du sein et quelqu'un vous rappellera pour répondre à vos questions / vous offrir du soutien.
- Si la personne refuse : Je vous invite à communiquer avec le médecin qui vous suit pour le cancer du sein ou votre infirmière-pivot. Si vous n'avez pas d'infirmière-pivot désignée, vous pouvez laisser un message à ce numéro XXXX et une infirmière vous rappellera.

À COMPLÉTER PAR L'INTERVIEWEUR SUITE À L'ENTREVUE

Observations sur le **déroulement** de l'entrevue (par exemple, ambiance, attitude la participante, éléments qui ont pu perturber l'entrevue, etc.)

____

____

____

Observation sur le **contenu** de l'entrevue (par exemple, les points importants qui ont été apportés par la femme, etc.)

____

____

____

Points à considérer pour les **prochaines entrevues** (par exemple, modifications à apporter au canevas, points qui doivent être abordés lors d'une prochaine entrevue, etc.)

____

____

____

Autres remarques :

____

____

____

GUIDE D'ENTREVUE INDIVIDUELLE POUR LES PROFESSIONELS
Projet Appli

SECTION A – INTRODUCTION

Vous avez une ou plusieurs patientes qui ont participé à une étude pilote portant sur l'évaluation d'une application permettant d'auto-rapporter les effets secondaires associés à un traitement contre le cancer en comprimés. Vous avez été invitée à participer à l'entrevue semi-dirigée de fin d'étude. Nous aimerions maintenant connaître votre expérience par rapport à ce projet. Votre expérience et vos opinions nous aideront à améliorer l'application. Je vous invite à exprimer librement ce que vous pensez.

Avec votre accord, cette entrevue sera enregistrée afin de faciliter l'analyse des données. La partie vidéo sera complètement détruite et seule la partie audio sera conservée sur un serveur informatique à accès restreint.

Avez-vous des questions avant qu'on ne commence?

UTILISATION

Premièrement, avez-vous vu dans le dossier de la patiente des rapports d'effets secondaires provenant de l'application?

- Si oui, passez à la question sur la facilité d'utilisation.
- Si non, demander quels ont été les barrières qui ont empêché la consultation/lecture de ces rapports.

SECTION B – APPRÉCIATION GLOBALE DE L'APPLICATION

Je vais maintenant vous poser des questions sur votre expérience avec l'ensemble des activités de l'application.

FACILITÉ D'UTILISATION

- **Quel a été votre expérience d'utilisation des rapports des effets secondaires de vos patientes?**
  - **Trouvez-vous que le rapport des effets secondaires était facile à comprendre?**
  - **Trouvez-vous que le rapport avait un impact sur le temps des visites médicales avec vos patients?**

UTILITÉ POUR LE SUIVI

- **Trouvez-vous que le rapport des effets secondaires améliorait votre suivi des patientes?**
  - **Si oui pourquoi?**
  - **Si non pourquoi?**

IMPLANTATION

- **Aimeriez-vous que l'utilisation de l'application soit implantée en pratique courante?**
  - **Facteurs facilitants?**

- **Barrières?**

APPRÉCIATION GLOBALE

- **Recommanderiez-vous l’utilisation de l’application en suivi des effets secondaires des traitements contre le cancer en comprimés à d’autres collègues?**

SECTION C – CONCLUSION

- **Avez-vous d’autres idées de ce qui pourrait être fait pour mieux accompagner et outiller les femmes qui reçoivent une prescription d’un traitement contre le cancer en comprimé suite à un cancer du sein?**

- **Y-a-t-il d’autres éléments que vous aimeriez apporter concernant votre expérience avec les patientes qui ont utilisé l’application?**

Je vous remercie beaucoup pour le temps que vous m’avez accordé aujourd’hui.
