# Supplementary material for: Development and Evaluation of a Web-Based App for Adverse Effect Management in Breast Cancer Patients Treated with Oral Targeted Therapy or Chemotherapy: Findings from a Pilot Study
Source: Curr Oncol. 2026 May 7;33(5):272. doi: 10.3390/curroncol33050272 (PMC13205787; doi:10.3390/curroncol33050272)
Supplement: Supplementary file 1 [file curroncol-33-00272-s001.zip › File S3 JCH.pdf]

SUPPLEMENTARY MATERIALS

QUOTES OF SEMI-STRUCTURED INTERVIEWS WITH WOMEN

Table. Quotes from the interviewed patients

Theme	Subtheme	Quotes
Using the App	1.1 Use initiation	1.1 *"Ben dans le fond, on me l'a proposé d'emblée quand on m'a proposé le médicament, fait que moi ça commencé en même temps, puis je l'ai utilisé comme ils demandaient de l'utiliser, à chaque fois, chaque jour." (Entrevue 4)*
		Well to be honest, it has been proposed to me at the same time as the medication, so for me, both started at the same time, and I used it as they instructed me to use it, each time, each day. (Interview 4)
	1.2 Day-to-day use	1.2 *"Ben moi, je l'ai utilisé tout le long. Je dis presqu'à tous les jours parce que les jours où il n'y avait pas de, parce qu'il y avait juste quatre symptômes identifiés journaliers, alors ces journées-là où il n'y avait pas lieu de répondre, ben on n'avait pas à répondre, mais je suis quand même allée voir à tous les jours là " (Entrevue 5)*
		Well me, I've used the whole time. I say almost every day because on the days when there was none, since there were only four symptoms identified daily, then on these days when there was nothing to answer, well we didn't have to answer. But I still went and checked every day. (Interview 5)
	1.3 Use over time	1.3*"Au début, la première semaine, je l'ai pas mal fait à tous les jours, et après ça, quand j'ai pris l'habitude d'aller sur mon téléphone, je le faisais quand j'avais un moment." (Entrevue 2)*
		At the beginning, the first week, and did it almost every day, and after that, when I got used to going on my phone, I went when I had the time. (Interview 2)
	1.4 Consultation of the instructions provided by the App	1.4 *"Dès que je les rapporte [les effets indésirables], tout dépendamment des réponses que je donne, on m'inscrit une stratégie, pas une stratégie mais des démarches à suivre en fait, selon les symptômes que j'ai rapportés." (Entrevue 6)*
		As soon as I report them (the side effects), depending on the answers I have given, it gives

Theme	Subtheme	Quotes
		me a kind of strategy, in fact not a strategy but steps to follow depending on the symptoms I reported. (Interview 6)
	1.5 Nature of the instructions provided by the App	1.5 *"Ben, en fait, la première fois là, j'ai vu ce que l'outil proposait, je suis allée voir sur mes feuilles, j'ai dit – Ah, c'est la même chose" (Entrevue 5)*
		Well, in fact, the first time I saw what the tool was proposing, I went to look at my papers and said - Ah, it's the same thing. (Interview 5)
	1.6 Calls to the healthcare professionals	1.6 " […] *quand je déclarais disons, parce que c'était le matin pour la veille, fait que quand je déclarais, souvent j'avais, quand je déclarais trop d'épisodes de diarrhées dans une journée, ben dans le fond, eux ils m'appelaient pour m'assurer que tout était correct, s'il fallait rajouter des médicaments, s'il y avait des choses à faire puis j'avais tout le temps un appel dans les 24h" (Entrevue 4)*
		[…] when I'd report, like, because it was the morning for the day before, so then when I would report, often if I would have, say, when I reported too many episode of diarrhea in the same day, well basically, they would call me to make sure everything was okay, if it was necessary to add drugs, if there was something that needed to be done then I would always get a call within 24 hours. (Interview 4)
Appreciation of the web-app	2.1 Overall performance	2.1 *"C'était facile, ça m'a fait plaisir de participer... tous les matins je répondais puis c'était facile. C'était pas dérangeant" (Entrevue 1).*
		It was easy, I was happy to take part… every morning I would answer and it was easy. It was not disrupting. (Interview 1)
	2.2 E-mail	2.2 *"Au départ, je m'attendais à avoir une appli sur mon téléphone... mais tu sais, ça marchait bien par courriel" (Entrevue 4).*
		At first, I expected to have an app on my phone… but you know, it was working well by email. (Interview 4)
	Frequency	2.3 *« Ben moi je trouve que c'est beaucoup. Mais c'est sûr que tu sais, moi, dans mon cas, c'était beaucoup parce que j'ai pas tant grand-chose, mais en même temps, quelqu'un qui feel vraiment pas, ben c'est sûr que c'est peut-être utile là. Oui, quelqu'un qui feel vraiment pas,*

Theme	Subtheme	Quotes
		ça va peut-être le rassurer de rentrer ses infos là-dedans, puis de peut-être recevoir un appel" (Entrevue 7) »
		Well my, I found that it was a lot. But it's true that, you know, for me, in my case, it was a lot because I do not have a lot, but at the same time, am someone who doesn't feel very well, really not, then it will reassure me to enter these info in that, then to maybe receive a call. (Interview 7)
	2.4 Treatment information	2.4 *"Oui, ben je les ai eus par courriel, je les ai eus physiquement, je les ai eus plein de fois parce que c'est un nouveau médicament là, mais tu sais, c'était bien fait, c'était super bien fait, c'était clair, c'était concis, c'était super bien fait." (Entrevue 4).*
		Yes, well I received them by email, I received them physically, I have gotten them a bunch of time because it is a new drug that, but you know, it was well done, it was very well done, was clear, was concise, was very well done. (Interview 4)
	2.5 Return from the healthcare professionals	2.5 *"Tout de suite, on m'a répondue tout de suite, tu sais, quand on est malade comme moi, ben des pilules c'est correct, c'est beau mais ce qu'on a besoin aussi c'est de soutien, ça j'en ai eu, ça m'a permis, ce sondage-là, d'avoir un soutien instantané […] Ils m'ont appelée tout de suite grâce au fond, grâce, c'est ça la patente là tu comprends." (Entrevue 1)*
		Immediately, they answered me right away, you know, when being sick like me, bunch of pills are correct, it's good, but what you also need is support, that I have gotten, it allowed me, this survey, to get instant support […] They called me right away thanks to, thanks, this is what this thing is you understand. (Interview 1)
	2.6 Reporting the adverse effects to a questionnaire instead than to a professional	2.6 a *"moi personnellement, c'est ça que j'aurais préféré qu'elle m'appelle. Je te dis pas m'appeler tous les jours, puis tu sais, ils sont tellement dans le jus aussi pauvres eux autres là. Mais tu sais peut-être de m'appeler une fois par mois, puis de dire – Hé, comment ça va les symptômes? Est-ce que c'est gérable? Est-ce que tu sais, on peut tu en jaser?" (Entrevue 7).*

Theme	Subtheme	Quotes
		2.6b *"[…] mes ampoules aux pieds, c'est pas arrivé en restant assis cette affaire-là, parce que j'ai exagéré, c'est moi qui a trop marché tu sais, fait que, c'est ça le questionnaire le demande pas tu sais, faut que tu parles à un être humain mais finalement, tu finis par parler à un être humain mais le sondage ne donne rien pour ça là, c'est vraiment l'humain à qui tu veux t'expliquer, écoute j'ai fait une folle de moi là, j'ai exagéré fait que" (Entrevue 1)*
		Me personally, that's what I would've preferred, that she called. I'm not saying to call me every day, then you know, they are so swamped too, poor them. But like, you know, maybe call me once a month, and then say – Hey, how are the symtoms? Is it manageable? Do you know? Do you wanna to talk about it? (Interview 7)<br>[…] the sores on my feet, it did not happen by staying setted this thing, because I exaggerated, it's me who walked too much you know, so then, this is what the questionnaire does not ask you know, you have to talk to a human being but in the end, the end up talking to a human being but the survey doesn't allow anything about that, it is really the human to who you want to explain this, listen I made a fool of myself then, I exaggerated so… (Interview 1)
Perceived impacts of using the application	3.1 A feeling of being supported and reassured	3.1 *"Ah ben moi, moi je me sentais suivie, je sentais qu'il y avait quelqu'un à l'écoute, surtout que j'avais des appels dans un délai très raisonnable quand il y avait quelque chose, fait que, non moi je me sentais bien là-dedans" (Entrevue 4).*
		Ah well me, I felt I was followed, I felt there was someone to listen, especially that I had calls in a very reasonable delay when there was something, so then, no I felt good in all of this. (Interview 4)
	3.2 Better management of side effects	3.2. *"Je pense pas que j'aurais appelé aussi souvent... ça aurait pris plus de temps avant que j'appelle, fait que ma condition aurait peut-être été, elle se serait peut-être empirée à ce moment-là" (Entrevue 4).*
		I do not think I would have called as often…. It would have taken more time before I called, so

Theme	Subtheme	Quotes
		my condition could have been, it could have become worse at that time. (Interview 4)
	3.3 Introspection	3.3. *"Elle [l'application] nous oblige à se questionner comment je vais aujourd'hui, donc, parce que des fois, on pense plus au train-train quotidien qu'à nous-même là, fait que c'est comme si, tu sais, ça dure pas longtemps, c'est 5 minutes puis bon – Aujourd'hui, j'ai tu mal? Ça t'oblige à réfléchir fait que donc, t'as moins de possibilité à mon avis que ça dégénère" (Entrevue 3).*
		It… (the app) force us to ask ourselves question about how I feel today, so, because some times, we think more about the daily routine then about ourselves, so then it's like that, you know, it does not last long, it's 5 minute than good – Today am I hurting? It compels you to think so then you have less possibility, in my opinion, for it to get out of hands. (Interview 3)
	3.4 Reminder of the disease	3.4 *"J'aurais aimé plus y aller de façon autonome, de cliquer sur un lien, puis d'aller mettre la date d'aujourd'hui... j'avais pas besoin d'un rappel d'aller sur le questionnaire […] C'est sécurisant d'inscrire tout ça, mais en même temps, pour moi, c'était un rappel qui était très confrontant par rapport à la maladie, par rapport à mon processus d'acceptation de la maladie. " (Entrevue 6)*
		I would have like to go for it in a more independent way, o click on a link, then to enter today's date… I did not need to have a reminder to go to the questionnaire […] It's reassuring to record all that, but a the same time, for me, it was the reminder regarding my disease and my process of accepting the disease that was very confronting. (Interview 6)
	3.5 No impact	3.5 *"Je me serais débrouillée avec ça parce que ce qui est important, c'est d'avoir des liens, des contacts... j'avais le email de la pharmacie, je l'avais, fait que je me serais débrouillée avec ça" (Entrevue 1)*
		I would have managed with this because what's important is having connections, contact… I had the pharmacy's email, I had it, so I would have managed with all that. (Interview 1)

Theme	Subtheme	Quotes
4 Confusion between apps and research activities	4.1 Weekly questionnaire	4.1 *"J'aurais aimé qu'il y aurait eu comme, parce que là, je pense qu'on parlait de nausées, diarrhées et fatigue [la femme fait référence au questionnaire journalier], mais j'aurais aimé ça qu'il y aurait eu un espace pour dire – Est-ce qu'il y a d'autres choses? Oui, mais bon, il l'avait au moins dans celui-là qui était hebdomadaire, fait qu'au moins je pouvais m'exprimer dans celui-là." (Entrevue 7)*
		I would have like it there would have been like, because now, I think we were talking about nausea, diarrhea and fatigue [The lady was referencing to the daily journal], but I would have like it if there'd been a space to say – Is there something else? Yes, but well, at least it was in the one that was weekly, so at least I could express myself in this one. (Interview 7)
	4.2 App intended solely for data collection for research	4.2 *"Définitivement, oui je le proposerais, oui, je dirais – Faut aider, participe, ça peut aider, je sais que pour vous autres, ça peut faire en sorte que mettons, 80 femmes qui ont eu des bobos dans la bouche, vous allez savoir." (Entrevue 1)*
		Definitely yes I would suggest it, yes, I would say – Must help, get involved, it can help, I know that for you guys, it can mean that, let's say, 80 women who have had mouth sores, you will know. (Interview 1)
5 Suggestions for improving the application	5.1 Human contact	5.1 *"Ce serait un contact, soit écrit, un petit mot écrit, ou par téléphone, sans que ce soit journalier ou hebdomadaire, au moins une fois dans le cycle." (Entrevue 2*)
		It'd be a contact, either in writing, a small written note, or by phone, without being daily or weekly, but at least once during the cycle. (Interview 2)
6 Expanded use of the App	6.1 Recommending the App to other women	6.1 *Intervieweure : "Puis pour quelle raison vous pensez que ça pourrait être bénéfique pour d'autres femmes?" Participante (P) : "(silence) Parce que c'est quand même un suivi. On se sent quand même liée même si c'est tenu comme fil (petit rire)" (Entrevue 2)*
		The Interviewer : « So for which reason you think it could be beneficial for other women? » Participant (P): (silence) Because it is still a follow-up. We still feel linked even if my a tread (small laugh). (Interview 2)

Theme	Subtheme	Quotes
	6.2 Use for other treatments	6.2 *"Moi je vous dirais, s'il y a d'autres médicaments qui ont des effets secondaires importants, je pense que ça serait bénéfique là [...] Ben je vous dirais que oui, puis tu sais, vous me faites penser quand j'étais en traitement de chimiothérapie, pendant que, dans le fond, la première chimio que j'ai eue, c'est peut-être des affaires qui peuvent être pertinentes aussi là, de savoir les effets secondaires qui sont après les traitements…" (Entrevue 3)*
		Me I would say, if there's other medications with important side effects, I think it would be beneficial then […] Well I'd tell you yes, then you know, you make me think when I was in chemo treatment, during, let's say, the first chemo I've had, it can be things that can be also relevant, to know the side effects happening after the treatments… (Interview 3)
	6.3 Utilisation prolongée dans le temps	6.3 *"D'ailleurs trois mois, je trouve que, d'après moi là, à moins que la personne soit très insécure mais trois mois, je trouve que c'est suffisant pour atteindre un niveau de confiance. Comme là, moi je me sens, c'est sûr que si jamais j'avais un problème, je sais que je peux appeler à la pharmacie mais j'ai pu besoin de compléter un questionnaire à tous les jours pour dire comment ça se passe, parce que là, après 3 mois, je trouve que c'est un bon délai pour prendre notre autonomie puis être confiante avec nos symptômes" (Entrevue 1)*
		Besides, three months, I think that, in my opinion, unless the person is very insecure but three months, I think it is enough to gain a level of trust. Like now, I feel that if I ever had a problem, I can call the pharmacy but I no longer need to complete the questionnaire every day to say how things are going, because then, after 3 months, I think it's a good period to become independent and confident about our symptoms. (Interview 1)

DETAILED RESULTS OF SEMI-STRUCTURED INTERVIEWS WITH WOMEN

1. Summary of the Approach

A total of seven interviews were conducted in the summer of 2023 with women who had completed their participation in the project. These women were identified by the research team (J-C Hogue) to represent different characteristics. The interviews were conducted by telephone (S Lauzier) using the interview guide presented in the appendix. The interviews were transcribed in full. A thematic analysis, assisted by NVivo software, was carried out by a research professional (A. Baghdadli) in collaboration with S Lauzier. This report presents the main findings of these interviews. For each identified theme and sub-theme, several excerpts from the interviews are provided as supporting evidence. The most relevant excerpts may be selected for publication.

2. Introduction to the application

Participants reported being introduced to the application primarily through virtual and telephone communication. The explanations provided covered how the application worked, the side effects of the medications involved, and how to report these effects through the application. One participant mentioned that she did not receive initial instructions, partly due to staff changes. Despite these challenges, the participant found the application relatively easy to use.

> *"Bon, quand on me l'a proposée, on m'a expliqué comment ça allait fonctionner. J'ai eu un appel aussi pour qu'on m'explique les effets secondaires et les effets indésirables de la molécule, le kisqali, et puis bon, ça s'est fait virtuellement, par informatique." (Entrevue 2)*

> *"Ben de la manière que je l'ai utilisée, puis c'est ça que je me suis posé la question, parce que j'ai eu comme des instructions verbales par téléphone, parce que l'infirmière qui s'occupait du projet quittait, fait qu'on a comme pas pu se parler [...] Je lui avais parlé au début parce qu'elle m'avait parlé un peu du projet puis j'avais dit que j'étais d'accord pour le poursuivre, mais les instructions, je les ai pas vraiment eues, mais bon, c'est assez facile d'application [...]" (Entrevue 3)*

> *"Ben dans le fond, on me l'a proposé d'emblée quand on m'a proposé le médicament, fait que moi ça commencé en même temps, puis je l'ai utilisé comme ils demandaient de l'utiliser, à chaque fois, chaque jour." (Entrevue 4)*

3. Use of the application

3.1. Start of use

Participants generally began using the application as soon as it was offered to them, i.e., at the start of treatment.

> *« Ben dans le fond, on me l'a proposé (l'application) d'emblée quand on m'a proposé le médicament, fait que moi ça commencé en même temps, puis je l'ai utilisé comme ils demandaient de l'utiliser, à chaque fois, chaque jour. » (Entrevue 4)*

> Interviewer: *"Est-ce que vous avez commencé à l'utiliser dès qu'on vous l'a recommandé ou vous avez attendu". Participante : "Dès que j'ai commencé le traitement, le produit, le ribociclib, au début oui Dès que j'ai commencé le traitement, le produit, le ribociclib, au début oui." (Entrevue 5)*

3.2 Use of Daily Symptom Reports

The reporting of adverse events following the sending of daily automated emails varied among women and throughout the study. For some, use was more regular at the beginning, often daily. However, over time, they adapted their use to suit their needs, responding less frequently according to their routine and the evolution of their symptoms. For example, some women did not respond daily on days when they did not experience significant symptoms or when they were busy or traveling. Despite this, most participants reported being diligent in their use of the application, responding almost every day throughout the study.

> *"En fait, c'était une fois par jour, je recevais un courriel, donc je devais ouvrir le courriel quand j'avais des symptômes à noter, c'est ça sur une base journalière." (Entrevue 6)*
>
> *"Ben dans le fond, on me l'a proposé d'emblée quand on m'a proposé le médicament, fait que moi ça commencé en même temps, puis je l'ai utilisé comme ils demandaient de l'utiliser, à chaque fois, chaque jour. Après ça, il y en avait un par semaine je pense" (Entrevue 4)*
>
> *"Au début, la première semaine, je l'ai pas mal fait à tous les jours, et après ça, quand j'ai pris l'habitude d'aller sur mon téléphone, je le faisais quand j'avais un moment." (Entrevue 2)*
>
> *"Ben ça bien été, c'était facile, ça m'a fait plaisir de participer, parce que je sais que ça peut aider des fois tu sais, alors non, puis tous les matins je répondais puis c'était facile. C'était pas dérangeant. […] j'ai probablement oublié pendant mes vacances […] fait que là je pense que j'en ai oublié un certain" (Entrevue 3)*
>
> *"Ben moi, je l'ai utilisé tout le long. Je dis presqu'à tous les jours parce que les jours où il n'y avait pas de, parce qu'il y avait juste quatre symptômes identifiés journaliers, alors ces journées-là où il n'y avait pas lieu de répondre, ben on n'avait pas à répondre, mais je suis quand même allée voir à tous les jours là " (entrevue 5)*

3.3. Use of recommendations provided by the application

The recommendations for managing side effects provided by the application were consulted when offered. For some women, they served as a reminder or supplement to the information provided (by the pharmacy) in the documentation at the start of treatment.

> *"Dès que je les rapporte [les effets indésirables], tout dépendamment des réponses que je donne, on m'inscrit une stratégie, pas une stratégie mais des démarches à suivre en fait, selon les symptômes que j'ai rapportés." (Entrevue 6)*
>
> Interviewer: *"Est-ce qu'en plus des communications avec l'équipe du Centre des maladies du sein, vous avez aussi utilisé les recommandations qui étaient incluses dans l'application ou ça été surtout avec les professionnels? "* Participante*: "Eh oui oui, je savais très bien ce qui était marqué dans, dans le fond, ce qu'ils nous disaient de faire dans l'application, ben c'était un repeat after me, parce que j'avais eu une feuille concernant ça quand j'ai eu mon enseignement*

sur cette fameuse pilule, on m'avait remis quoi faire si au cas où que, fais ci, fais ça tu sais, ça revenait pas mal au même là tu sais, ben ça revenait au même." (Entrevue 1)

Interviewer : *"Puis vous dans la gestion de vos nausées ou problèmes digestifs, qu'est-ce qui a été le plus déterminant pour trouver une solution? […] est-ce que ça venait plutôt de votre lecture du feuillet, de la recommandation de l'outil ou de votre discussion avec le médecin? Qu'est-ce qui vous a permis de régler ce problème-là le mieux? "* Participante *: "Mais en fait, la nausée c'était très léger comme je dis, ça c'est juste avec les feuillets ou avec, en fait, ce qui l'a réglé le problème de la nausée, c'est quand on a réglé le problème de la digestion. Ça, c'est avec mon médecin. […] En fait, c'est les mêmes recommandations que j'avais sur mes feuilles d'effets secondaires possibles […] " (Entrevue X)*

Interviewer : *"Puis vous, qu'est-ce que vous avez consulté? Vos feuilles ou ce qui était proposé par l'outil? "* Participante *: "Ben, en fait, la première fois là, j'ai vu ce que l'outil proposait, je suis allée voir sur mes feuilles, j'ai dit – Ah, c'est la même chose" (Entrevue 5)*

"On me demandait d'être vraiment à l'affût si les symptômes perduraient, de mettre de la glace, de faire attention" (Entrevue 6).

3.4 Receiving calls from the care team

Of the 7 women interviewed, 5 reported receiving feedback from the care team after completing a daily questionnaire. Patients were contacted promptly (within 24 hours) after reporting a symptom to assess its severity and suggest medication adjustments if necessary.

"Ben dans le fond, quand je cochais mes, dans le fond, mes symptômes, quand je les cochais, ben tu sais, j'ai eu des appels, fait que ça m'a évité moi d'avoir à appeler, parce que tu sais, quand ils nous donnent le médicament, on a des, disons si tel symptôme apparait, tel symptôme apparait, on a des actions à faire, puis en ayant cette application-là, moi ça m'a permis de pas avoir à appeler. Eux, ils m'appelaient avant que je les appelle, fait que […] quand je déclarais disons, parce que c'était le matin pour la veille, fait que quand je déclarais, souvent j'avais, quand je déclarais trop d'épisodes de diarrhées dans une journée, ben dans le fond, eux ils m'appelaient pour m'assurer que tout était correct, s'il fallait rajouter des médicaments, s'il y avait des choses à faire puis j'avais tout le temps un appel dans les 24h" (Entrevue 4)

" […] quand il y avait des nausées, mais c'était très léger comme nausées. Mais je les ai quand même mentionnées, une fois que ça été un petit peu plus fort puis on m'a appelée tout de suite, ça, j'ai trouvé ça très bien. J'ai été même agréablement surprise que ça soit si rapide la réponse à ça." (Entrevue 5)

3.5 Weekly Questionnaire

Beyond the daily functions offered by the application, the women mentioned that the weekly questionnaire was used to report symptoms or adverse effects not covered by the daily questionnaire. Participants appreciated this questionnaire for its ability to gather detailed information about their health status over time.

"En fait, pour rapporter mes effets indésirables, c'était plus sur l'hebdomadaire, une fois semaine, le questionnaire une fois semaine qui n'était pas le même que journalier là. Le journalier, le seul effet où j'ai répondu c'est quand il y avait des nausées, mais c'était très léger comme nausées. Mais je les ai quand même mentionnées, une fois que ça été un petit peu plus fort puis on m'a appelée tout de suite, ça, j'ai trouvé ça très bien." (Entrevue 5)

"J'aurais aimé qu'il y aurait eu comme, parce que là, je pense qu'on parlait de nausées, diarrhées et fatigue [la femme fait référence au questionnaire journalier], mais j'aurais aimé ça qu'il y aurait eu un espace pour dire – Est-ce qu'il y a d'autres choses? Oui, mais bon, il l'avait au moins dans celui-là qui était hebdomadaire, fait qu'au moins je pouvais m'exprimer dans celui-là." (Entrevue 7)

"Mais dans le hebdomadaire je le rapportais. J'ai rapporté des maux de dos, tu sais, dans le bas et tout, mais ça c'est comme normal. Mais je les ai rapportés quand même, étant donné que c'est des métastases aux os, j'imagine que le mal était normal, mais je le rapportais quand même le mal dans le bas du dos, au niveau des reins, mais c'est plus le bassin là puis les hanches, que j'ai rapporté." (Entrevue 7)

4. App Component Assessment

Users expressed overall satisfaction with the app's functionality. The app offered a simple, non-intrusive, and user-friendly daily experience for the majority of women. It was considered very easy to use, and the instructions were clear. This ease of use was highlighted as a strength of the app, contributing to a positive user experience. Some users reported quickly adapting to regular use of the app, integrating its features into their daily routine.

"C'était facile, ça m'a fait plaisir de participer... tous les matins je répondais puis c'était facile. C'était pas dérangeant" (Entrevue 1).

"Au début, la première semaine, je l'ai fait tous les jours, et après ça, quand j'ai pris l'habitude... je le faisais quand j'avais un moment" (Entrevue 2).

"C'était vraiment facile, oui, j'ai pas eu de problème avec ça" (Entrevue 7).

"Tous les jours, j'allais voir mon petit courriel, puis le lundi, je recevais celui-là hebdomadaire, fait qu'il n'y avait rien de compliqué là" (Entrevue 7).

The use of email as the application's primary communication channel elicited a range of reactions, often influenced by users' preconceived expectations. Some participants demonstrated adaptability to this unexpected but ultimately functional format. For other participants who prefer traditional communication methods, email was particularly appreciated. One woman, however, anticipated that using email might pose a challenge for some, even though she herself found it easy to use.

"Au départ, je m'attendais à avoir une appli sur mon téléphone... mais tu sais, ça marchait bien par courriel" (Entrevue 4).

"Pour moi, je suis de la vieille école, fait que le courriel c'est encore à la mode dans mon cas" (Entrevue 5).

"Pour une personne comme ma mère qui a 72 ans, bien, elle, tu pourras pas la rejoindre mettons avec un courriel, ça c'est sûr et certain" (Entrevue 7).

The frequency of email reminders was generally well-received. However, it was criticized by some users who would have preferred a more autonomous approach. For three participants, adapting to the reminder frequency seemed to vary over time, with initial acceptance decreasing as they became more accustomed to the application. For example, one user expressed that daily reminders are not always necessary, especially in the absence of significant symptoms. Another mentioned that the lack of autonomy and flexibility when using the application was burdensome and constantly reminded her of her illness. One participant suggested that the frequency of requested interactions could be perceived as intrusive, repetitive, and overwhelming, especially if users do not have new symptoms to report regularly. The participants also discussed the frequency of interactions with the application. While some found the daily frequency reassuring and a sign of attention, others considered it repetitive, especially when they did not experience symptoms requiring frequent reporting.

Interviewer *: "Puis comment vous avez trouvé la fréquence des courriels qui vous étaient envoyés pour répondre au sondage, à chaque jour? [...]"* Participante *: "Ben je trouvais ça correct, ça me dérangeait pas." (Entrevue 1)*

"J'aurais aimé plus y aller de façon autonome, de cliquer sur un lien, puis d'aller mettre la date d'aujourd'hui... j'avais pas besoin d'un rappel d'aller sur le questionnaire [...] C'est sécurisant d'inscrire tout ça, mais en même temps, pour moi, c'était un rappel qui était très confrontant par rapport à la maladie, par rapport à mon processus d'acceptation de la maladie. " (Entrevue 6).

"Je trouvais ça très, très lourd de recevoir un courriel par jour par rapport à ça... j'aurais pu juste avoir un lien dans mes favoris [...] J'aurais aimé plus y aller de façon autonome... parce que tous les jours, je le sais que je prends un traitement tous les jours" (Entrevue 6).

"La fréquence une fois par jour pour moi, je trouvais ça trop" (Entrevue 6).

" (...) moi dans le fond, c'était vraiment en début de traitement, fait qu'il y avait un traitement qui est encore ''nouveau", fait que c'était important de bien suivre les symptômes, parce qu'ils peuvent être gros, fait que dans le moment, ben tu sais, si je l'avais encore aujourd'hui, ben peut-être que je serais tannée de le remplir une fois par jour. " (Entrevue 4).

"Ben le journalier, je trouvais, à tous les jours, c'était comme toujours pareil, les quatre mêmes, tu sais, comme le fait justement qu'on pouvait pas, si on a de quoi qui nous chicote qui n'est peut-être pas grave, ou qui n'est pas dans les symptômes hebdomadaires ou quoi que ce soit, tu sais qui était journaliers qu'on aurait pu ajouter tu sais, pour le mentionner là, mais sinon, mais peut-être le fait que j'avais pas les symptômes qui étaient décrits là, à part une petite nausée, pour moi, c'était comme, je le remplissais parce que c'était pas long à remplir, ça c'était la bonne chose là. Mais mettons que ça aurait été tous les jours les quatre symptômes, puis avoir à répondre à chacun des symptômes avec des catégories un petit peu, un peu plus, un peu plus. Si ça avait été plus long, ça m'aurait, j'aurais trouvé ça un peu plate dans le sens où si ça s'applique pas, ben ça te tente pas de répondre à des questions qui s'appliquent pas à toi là tu sais. Mais là, le fait que c'était court, léger, puis si t'avais aucun de ces symptômes-là, t'as

juste à pas répondre. Il y a juste que pour vous autres, je me dis – Si je réponds pas, est-ce qu'ils savent que je suis allée sur le site" (Entrevue 5)

Interviewer : *"Puis qu'est-ce que vous avez pensé de la fréquence là, du fait que c'était envoyé une fois par jour? "* Participante : *"Ben moi je trouve que c'est beaucoup. Mais c'est sûr que tu sais, moi, dans mon cas, c'était beaucoup parce que j'ai pas tant grand-chose, mais en même temps, quelqu'un qui feel vraiment pas, ben c'est sûr que c'est peut-être utile là. Oui, quelqu'un qui feel vraiment pas, ça va peut-être le rassurer de rentrer ses infos là-dedans, puis de peut-être recevoir un appel" (Entrevue 7)*

Users expressed overall satisfaction with the clarity and quality of the documentation provided by the application, as well as its usefulness in addressing reported symptoms in a personalized way. A comparison between digital and traditional (paper) documentation revealed a preference for the digital format due to its instant interaction, according to one participant. However, some users also appreciated physical documents for their tangible nature and potential accessibility at any time. The reminders and practical advice included in the documentation were highly valued, particularly those related to managing side effects.

"Ben moi je dirais pour moi, étant donné que j'avais, la plupart des symptômes, j'avais la version écrite des effets secondaires possibles à différents niveaux, puis que c'est quelque chose que je consultais régulier, fait que pour moi ça peut-être pas fait l'effet que ça pourrait faire sur quelqu'un qui n'a pas les papiers [...] Oui, ça, je les ai, je dors pas avec, c'est pas sur ma table de chevet mais non, je les ai toujours près, sont avec mes autres papiers de rendez-vous et ces choses-là, fait que si j'ai un questionnement ou quelque chose, je vais voir sur, je vais consulter les papiers" (Entrevue 5)

"Oui, ben c'est ça. En fait, dès que je les rapporte, tout dépendamment des réponses que je donne, on m'inscrit une stratégie, pas une stratégie mais des démarches à suivre en fait, selon les symptômes que j'ai rapportés. Donc c'est sûr que c'est quand même assez personnalisé, puis si on voit que les symptômes sont plus importants, ben là, ils me demandent d'appeler ou de cesser la médication. Donc, il y a quand même un suivi là-dessus qui est fait de façon personnalisée, mais en même temps, on avait déjà reçu un document assez détaillé là sur papier, qui nommait un peu les mêmes choses. Seulement lui c'était plus interactif en répondant automatiquement, tandis que la version papier ben c'était comme moi, un petit peu à cocher puis à voir où est-ce qu'on en est rendu. Donc c'est ça [...] Les recommandations étaient très appropriées puis j'ai pu appliquer celles qui s'adaptaient à ma situation, mais c'était pour moi des rappels tout ça [...] C'était parfait, ah oui, vraiment, c'était très, très adapté. Puis pour moi, c'était vraiment, mais vraiment clair. J'aurais aimé avoir des outils comme ça pour les autres traitements que j'ai eus, que je trouvais que j'étais vraiment plus dans le néant" (Entrevue 6)

"[...] la feuille, elle était vraiment belle, elle était en couleur (rires), puis tu sais, moi ça m'a accroché l'œil, fait que je l'avais vraiment plus en tête. [...] le programme lui, c'était sur ordinateur, c'était en noir et blanc, puis j'ai pas pu l'apprendre tu sais, j'ai pas été capable de le maîtriser" (Entrevue 6)

"Oui, ben je les ai eus par courriel, je les ai eus physiquement, je les ai eus plein de fois parce que c'est un nouveau médicament là, mais tu sais, c'était bien fait, c'était super bien fait, c'était clair, c'était concis, c'était super bien fait." (Entrevue 4).

Regarding the daily questionnaires, participants found their daily completion reassuring, providing a sense of support and relevant guidance throughout their treatment experience. The women's experience was enhanced by the system's responsiveness and the support they perceived through interactions with the care teams. Follow-up calls in response to questionnaire entries were particularly appreciated, giving users the feeling of being listened to and proactively cared for. One participant mentioned that completing the questionnaire allowed her to reflect daily on her health and the onset of any side effects, giving her the opportunity to react early and quickly to manage potential symptoms.

Participants appreciated the healthcare team's prompt and appropriate response when they reported certain side effects, without them having to initiate any action themselves. On the other hand, the rigidity of the questionnaires and the lack of personalization and contextualization of symptoms have been criticized. Women who did not experience specific symptoms sometimes felt the interaction was irrelevant.

"Eh, s'il y avait eu un endroit pour indiquer – Je n'ai pas d'effets secondaires ni d'effets indésirables, ceux qui étaient cités, les quatre dans le questionnaire quotidien" (Entrevue2).

"Mais mettons qu'on va juste avec votre outil, parce que c'est un peu ça. Ben j'ai quand même trouvé que c'est un bel accompagnement de dire – Bon ben qu'à tous les jours, pour quelqu'un ça peut être rassurant d'avoir cet accompagnement-là, puis cet outil-là. Au début, je trouvais ça vraiment intéressant, puis c'est ça, tu sais, finalement, avec les jours qui passaient, je me dis – Bon ben, j'ai pas de symptôme, fait que c'est plus ou moins utile. Après ça, ben c'est ça, la lourdeur s'est installée mais sinon, au départ départ, j'ai fait – Wow, c'est vraiment intéressant là. Parce qu'on me nommait que l'infirmière voyait les résultats automatiquement, pas l'infirmière mais la pharmacie, puis qu'ils m'appelaient, je trouvais ça vraiment proactif là, voilà" (Entrevue 6)

Interviewer *: "[...] cette application-là, elle sert à quoi également, qu'est-ce qu'elle apporte vous croyez? Qu'est-ce qu'elle vous a apporté à vous dans votre expérience avec le médicament?"* Participante *: "Elle nous oblige à se questionner comment je vais aujourd'hui, donc, parce que des fois, on pense plus au train-train quotidien qu'à nous-même là, fait que c'est comme si, tu sais, ça dure pas longtemps, c'est 5 minutes puis bon – Aujourd'hui, j'ai tu mal? Ça t'oblige à réfléchir fait que donc, t'as moins de possibilité à mon avis que ça dégénère. Je dirais que c'est ça le plus là."* (Entrevue X)

Interviewer : "J'aimerais savoir votre avis sur l'application, donc vous m'avez dit des choses comme – C'était rassurant, donc qu'est-ce qui était rassurant pour vous dans l'utilisation de cette application-là, ce sondage-là? Qu'est-ce qui a été le plus rassurant? Les éléments de l'application qui vous ont permis de vous rassurer? Participante *"Ben c'est qu'en écrivant comme ça à tous les matins, tous les matins dans le fond c'est comme si on s'informait de moi, alors dès que ça n'allait pas bien, ça été juste ça, un petit peu le bout des doigts écorchés, ça faisait en tout cas, mais tout de suite on, c'est la rapidité tu sais avec laquelle on me répondait, ouin" (Entrevue 1)*

"Tout de suite, on m'a répondue tout de suite, tu sais, quand on est malade comme moi, ben des pilules c'est correct, c'est beau mais ce qu'on a besoin aussi c'est de soutien, ça j'en ai eu, ça m'a permis, ce sondage-là, d'avoir un soutien instantané […] Ils m'ont appelée tout de suite grâce au fond, grâce, c'est ça la patente là tu comprends." (Entrevue 1)

"Ben dans le fond, quand je cochais mes, dans le fond, mes symptômes, quand je les cochais, ben tu sais, j'ai eu des appels, fait que ça m'a évité moi d'avoir à appeler, parce que tu sais, quand ils nous donnent le médicament, on a des, disons si tel symptôme apparait, tel symptôme apparait, on a des actions à faire, puis en ayant cette application-là, moi ça m'a permis de pas avoir à appeler. Eux, ils m'appelaient avant que je les appelle, fait que." (Entrevue 4)

"Le journalier, le seul effet où j'ai répondu c'est quand il y avait des nausées, mais c'était très léger comme nausées. Mais je les ai quand même mentionnées, une fois que ça été un petit peu plus fort puis on m'a appelée tout de suite, ça, j'ai trouvé ça très bien." (Entrevue 5)

"La seule chose que je vois de pratique, que j'ai trouvé de pratique là-dedans, c'est que, tu sais, si ça allait mal, tout de suite la pharmacie regardait ça le matin, les réponses de sondage, puis tout de suite j'ai été appelée." (Entrevue 1).

"Ben le journalier, je trouvais, à tous les jours, c'était comme toujours pareil, les quatre mêmes, tu sais, comme le fait justement qu'on pouvait pas, si on a de quoi qui nous chicote qui n'est peut-être pas grave, ou qui n'est pas dans les symptômes hebdomadaires ou quoi que ce soit, tu sais qui était journaliers qu'on aurait pu ajouter tu sais, pour le mentionner là, mais sinon, mais peut-être le fait que j'avais pas les symptômes qui étaient décrits là, à part une petite nausée, pour moi, c'était comme, je le remplissais parce que c'était pas long à remplir, ça c'était la bonne chose là. Mais mettons que ça aurait été tous les jours les quatre symptômes, puis avoir à répondre à chacun des symptômes avec des catégories un petit peu, un peu plus, un peu plus. Si ça avait été plus long, ça m'aurait, j'aurais trouvé ça un peu plate dans le sens où si ça s'applique pas, ben ça te tente pas de répondre à des questions qui s'appliquent pas à toi là tu sais. Mais là, le fait que c'était court, léger, puis si t'avais aucun de ces symptômes-là, t'as juste à pas répondre. Il y a juste que pour vous autres, je me dis – Si je réponds pas, est-ce qu'ils savent que je suis allée sur le site" (Entrevue5)

5. The Most and Least Liked Aspects of the App

5.1 Most Liked Features of the App

Participants found the app easy to use. They appreciated the simplicity of the interface and the ease with which they could answer daily questions. Participants greatly appreciated the ongoing follow-up after reporting side effects, feeling closely monitored and reassured about the medication.

"Mais globalement, comme je dis, ce que j'ai apprécié ça facilité l'utilisation de ça, ramener les choses au plus simple, pas compliquer les choses, ça c'est apprécié. " (Entrevue 5)

"Ben j'ai aimé que c'était facile, j'ai aimé que c'était rapide, tu sais, ça m'a pas dérangée parce que c'était pas quelque chose, c'était pas long à remplir là, bing bang boung, puis c'était fini." (Entrevue 1)

"C'est facile, j'ai trouvé ça très très facile, très convivial, très facile." (Entrevue 3)

"Le sentiment qu'il y a quelqu'un au bout de la ligne qui me fait le suivi, qui me surveille, qui surveille que je vais bien, fait que c'est sûr que ça donne un sentiment de confiance." (Entrevue 3)

"Ben c'est le suivi, c'est vraiment le fait d'avoir l'impression d'avoir un bon suivi puis de, ben je dis l'impression, mais j'en ai eu un bon là, c'est vraiment un encadrement, je trouve que c'est vraiment une plus-value." (Entrevue 4)

5.2 Least Popular Features of the Application

Some users found the experience robotic and impersonal, lacking human interaction, which made using the application less enjoyable. The limited options for reporting specific information or particular contexts were a less popular point for some users, highlighting a lack of flexibility in the questionnaires.

"*Ben moi je trouve que ce type de sondage-là, c'est un peu comme si on parlait à un robot han, c'est les mêmes questions à tous les jours, les mêmes réponses [...] Le sondage c'est des questions robotiques puis c'est ça.* " (Entrevue 1)

"*moi personnellement, c'est ça que j'aurais préféré qu'elle m'appelle. Je te dis pas m'appeler tous les jours, puis tu sais, ils sont tellement dans le jus aussi pauvres eux autres là. Mais tu sais peut-être de m'appeler une fois par mois, puis de dire – Hé, comment ça va les symptômes? Est-ce que c'est gérable? Est-ce que tu sais, on peut tu en jaser?*" (Entrevue 7).

"[...] mes ampoules aux pieds, c'est pas arrivé en restant assis cette affaire-là, parce que j'ai exagéré, c'est moi qui a trop marché tu sais, fait que, c'est ça le questionnaire le demande pas tu sais, faut que tu parles à un être humain mais finalement, tu finis par parler à un être humain mais le sondage ne donne rien pour ça là, c'est vraiment l'humain à qui tu veux t'expliquer, écoute j'ai fait une folle de moi là, j'ai exagéré fait que" (Entrevue 1)

Two participants reported no significant negative aspects in their experience using the application.

"Non, j'ai eu une très belle expérience, fait que j'ai pas, heureusement client satisfait a souvent moins de choses à dire qu'un client insatisfait" (Entrevue4).

6. Perceived Impacts of Using the Tool

6.1 Support and Reassurance

Most participants felt supported and reassured by the information provided and the regular use of the application, which allowed them to monitor the progress of their condition and feel supported by a medical team. This constant virtual presence was perceived as reassuring and encouraging. Generally, participants felt reassured by the support and guidance they received.

Interviewer : *"Puis quand vous parlez de bien-être, qu'est-ce que ça vous apportait d'utiliser ce questionnaire-là?"* Participante *: "Ben en fait, chaque fois qu'on, moi j'appelle ça, chaque*

médicament a sa personnalité, fait que chaque fois qu'on commence un nouveau médicament, ben il y a des nouveaux symptômes qui apparaissent avec lui, puis on les connait pas au début. Fait que c'est sûr qu'on est toujours un petit peu craintive à chaque fois qu'on commence un nouveau médicament […] on est un peu devant l'inconnu là de comment ça va se passer. Donc en étant assurée d'être soutenue par une équipe, ben c'est sûr que ça nous donne confiance de prendre le médicament puis que, ensemble, on va, ben faire l'expérience de ce médicament-là puis trouver des solutions, donc si c'était dit aux patientes que ça va aider à trouver la bonne dose, à s'assurer que elle va pas dépasser la limite qui fait que ça va devenir très difficile supposons à marcher effectivement, sa condition va devenir difficile puis que ça va éventuellement, ben poser un certain handicap temporaire par la douleur puis par, je pense que naturellement c'est ça que tu veux là, avoir du soutien puis explorer ce nouveau médicament-là avec quelqu'un expérimenté. […]" (Entrevue 3)

"Comment je me suis sentie? C'est sécurisant? Ouin, c'est sécurisant" (Entrevue 6)

"J'ai vraiment trouvé que c'était un bel accompagnement personnalisé. Contrairement aux autres traitements que j'ai eus, j'avais eu deux traitements avant celui-là, puis mon doux, j'avais jamais eu un aussi bel accompagnement puis aussi bien préparée que ça" (Entrevue6).

"Ah ben moi, moi je me sentais suivie, je sentais qu'il y avait quelqu'un à l'écoute, surtout que j'avais des appels dans un délai très raisonnable quand il y avait quelque chose, fait que, non moi je me sentais bien là-dedans" (Entrevue4).

"ben le sentiment qu'il y a quelqu'un au bout de la ligne qui me fait le suivi, qui me surveille (rires), qui surveille que je vais bien (rires), fait que c'est sûr que ça donne un sentiment de confiance […]" (Entrevue 4)

6.2 Effective Communication Tool for Optimizing Adverse Effect Management

Participants who had a positive experience with the application noted improvements in symptom management thanks to more proactive and personalized follow-up from the healthcare team. The tool was perceived as an effective way to communicate quickly and efficiently with healthcare professionals, which potentially prevented the worsening of symptoms.

"Je pense pas que j'aurais appelé aussi souvent... ça aurait pris plus de temps avant que j'appelle, fait que ma condition aurait peut-être été, elle se serait peut-être empirée à ce moment-là" (Entrevue4).

"Oui parce que ça peut être utile en cas d'urgence, parce que à ce moment-là, si t'as de quoi, ils vont t'appeler tout de suite grâce au sondage" (Entrevue1).

"[…] la seule chose que je vois de pratique, que j'ai trouvé de pratique là-dedans, c'est que, tu sais, si ça allait mal, tout de suite la pharmacie regardait ça le matin, les réponses de sondage, puis tout de suite j'ai été appelée, mais tu sais, j'ai pas été malade tant que ça là tu sais, mais quand je l'ai été, c'est sûr ils m'ont appelée tout de suite grâce au fond, grâce, c'est ça la patente là tu comprends" (Entrevue 1)

"Eh, je le sais pas, je pense pas que j'aurais appelé aussi souvent, je suis une personne qui n'aime pas déranger, qui aime m'arranger, fait que probablement que j'aurais pas appelé si souvent à la pharmacie que des fois qu'ils m'ont appelée, fait que peut-être que ça se serait régularisé moins rapidement" (entrevue4).

"Je trouvais ça bien que ça existe [...]. Je trouvais ça bien qu'il y ait ce lien quand même. [...] c'était un outil que j'étais capable d'utiliser facilement. C'est toujours le, c'est ça, puis le fait de pas avoir aller à l'hôpital à chaque fois, [...] c'est bien, je pense que ça vaut la peine de poursuivre puis raffiner l'outil" (Entrevue 2)

One participant, however, reported some uncertainty as to whether the application had contributed to faster care or significantly improved communication with the care team.

"[...] Le sondage, ça permettait que, c'était comme une ligne directe grâce au sondage tu sais, au début là, comprends tu? Mal aux pieds, ben là le sondage me servait à rien, pas du tout, je veux dire, ce qui m'aidait c'était vraiment, c'était monstrueux, c'était gros, ben là, on a travaillé ça par email, par téléphone, c'était pas avec le sondage [...] J'ose espérer que le sondage m'a permis d'avoir des contacts humains plus rapidement... mais je me suis aperçu un peu plus tard... pourquoi, ils sont compétents bien sûr, mais tu sais, comme je t'ai dit, tout le monde savait que j'étais en Floride, puis tout le monde savait que j'avais pas d'assurance [...] puis ils me l'avaient dit aussi – Madame [nom retiré], quand vous allez être partie, si vous avez besoin, tout de suite on va vous répondre, tout de suite" (Entrevue 1)

6.3 Enables introspection

One participant mentioned that using the app allows for daily introspection on the treatment experience and its progress. This enables more proactive monitoring of symptoms and faster action based on their evolution.

"Elle nous oblige à se questionner comment je vais aujourd'hui, donc, parce que des fois, on pense plus au train-train quotidien qu'à nous-même là, fait que c'est comme si, tu sais, ça dure pas longtemps, c'est 5 minutes puis bon – Aujourd'hui, j'ai tu mal? Ça t'oblige à réfléchir fait que donc, t'as moins de possibilité à mon avis que ça dégénère" (Entrevue 3).

"[...] c'était bien d'avoir le résumé de la semaine, à chaque semaine, on recevait un résumé, puis là on pouvait mettre, si on avait d'autres symptômes, ça j'ai trouvé que c'était quand même bien parce que ça nous porte à faire un rappel à, nous-même faire un petit rappel de comment notre semaine s'est passée [...]" (Entrevue 3)

"C'est tellement facile à répondre, ça prend 3 minutes, on peut tellement perdre du temps dans notre vie à faire plein d'autres affaires, ça j'ai jamais eu le sentiment que c'était inutile parce que comme je vous dis, ça m'obligeait aussi à me dire – Bon ben à matin, as-tu mal? Comment ça va à matin? On le fait pas toujours parce que des fois, on s'occupe plus des autres que nous-même" (Entrevue 3)

6.4 Constant Reminder of the Illness

A less positive effect noted was the feeling of burden and the regular confrontation with the illness due to the application's frequent reminders. This was expressed by one participant as a painful reminder of her condition, sometimes generating sadness and anxiety. This participant described the burden both in technical terms, as some functions were not optimal, and also in terms of the fact that it reminded her of her illness daily, hindering her process of accepting it.

> *"C'est sécurisant d'inscrire tout ça, mais en même temps, pour moi, c'était un rappel qui était très confrontant par rapport à la maladie, par rapport à mon processus d'acceptation de la maladie[...]* Interviewer: "*Puis quand vous dites que c'était très confrontant, qu'est-ce que ça suscitait comme réactions, émotions pour vous?"* Participante *: "Eh, de la tristesse, de l'inquiétude, puis du déni (rires), ouin c'est ça" (Entrevue 6).*

> *"Oui, c'est ça, ça me confrontait par rapport à ma maladie comme tout le temps là, fait que c'est ça, c'est vraiment au niveau psychologique, au niveau personnel." (Entrevue 6)*

6.5 Improved Treatment Awareness

The app encouraged users to be more aware of and attentive to their daily health. Regularly completing the questionnaires helped them to actively reflect on their health status and take proactive steps in case of new symptoms or side effects.

> *"Ben en fait, chaque fois qu'on, moi j'appelle ça, chaque médicament a sa personnalité, fait que chaque fois qu'on commence un nouveau médicament, ben il y a des nouveaux symptômes qui apparaissent avec lui, puis on les connait pas au début" (Entrevue3).*

> *"Ça été vraiment là, tu sais, souvent c'est subjectif voir les symptômes, je trouve ça difficile. Tandis que là, en ayant mettons, douleurs importantes ou douleurs moins importantes, ben je pouvais voir les interventions entre les deux, puis pouvoir mieux intervenir dans ma situation puis dire – Ok, je prends telle intervention parce que je trouve qu'effectivement, elle est adaptée ou pas celle-là tu sais" (Entrevue 6)*

6.6 Feeling of Helping Other Women Through Research

Some participants believed that completing the questionnaire was solely for research purposes. By completing the questionnaires, they felt they were actively contributing to research that could help other women taking this type of medication. They see the tool as potentially useful for improving data and research on treatments, and they would recommend its use in this context, even if the benefit would not be direct.

> *"Définitivement, oui je le proposerais, oui, je dirais – Faut aider, participe, ça peut aider, je sais que pour vous autres, ça peut faire en sorte que mettons, 80 femmes qui ont eu des bobos dans la bouche, vous allez savoir."* (Entrevue 1)

> *"Ben ça bien été, c'était facile, ça m'a fait plaisir de participer, parce que je sais que ça peut aider des fois tu sais, alors non, puis tous les matins je répondais puis c'était facile. C'était pas dérangeant."* (Entrevue1)

> *"[…] j'ai trouvé ça correct, ça m'a pas dérangée là. C'était pas une corvée là […] c'est une étude, on répond à l'étude (petit rire), si je m'engage, habituellement quand je m'engage dans quelque chose c'est parce que je le veux bien, c'est personne qui me force la main, c'est que habituellement, je vais au bout […]"* Interviewer*: "Donc c'est pas, c'est ça, dans votre expérience du traitement, vous, vous avez accepté de participer à une étude, vous vous étiez engagée comme vous dites, vous aviez envie de le faire pour les besoins de l'étude.[…]"* Participante : *"C'est pas un outil qui va qui m'aider avec la maladie là […] Dans le fond, c'est un outil pour vous aider, vous autres, à comprendre les médicaments j'imagine, qu'est-ce que les gens ont comme, à faire quasiment un sondage sur les effets secondaires là, tu sais, ce que moi j'en ai pensé" (Entrevue 5)*

6.7 Little or no perceived impact

Three participants indicated that, although they were willing to use the tool to support research, it did not change their personal experience of treatment. They mentioned that they would have managed their medical situation without the tool in the same way they did with it. They emphasized that, while the act of reporting was reassuring, it did not change the intensity of their symptoms or lead to any specific medical intervention.

> *"Je me serais débrouillée avec ça parce que ce qui est important, c'est d'avoir des liens, des contacts... j'avais le email de la pharmacie, je l'avais, fait que je me serais débrouillée avec ça"* (Entrevue 1)

> *"Le mentionner, ça n'a pas fait de différence. Et puis ça durait, l'intensité était pas tellement forte non plus. Ça m'inquiétait pas outre mesure, dans un sens, je vous dirais quasiment – J'étais bien contente que ce soit juste ça, parce que c'est important, je pense que c'est important, très important de le faire, mais que je n'avais pas d'inquiétude outre mesure, alors que personne m'appelle pour me dire – Bon ben vous avez ça, ça, ça, est-ce que ça s'est intensifié? Est-ce que, non, non."* (Entrevue2).

> *"Ben moi je dirais pour moi, étant donné que j'avais, la plupart des symptômes, j'avais la version écrite des effets secondaires possibles à différents niveaux, puis que c'est quelque chose que je consultais régulier, fait que pour moi ça peut-être pas fait l'effet que ça pourrait faire sur quelqu'un qui n'a pas les papiers."* (Entrevue5)

7. Three-Word Description of the Application

To understand the participants' overall perception of the application and identify its most salient features, we asked four participants to describe it in three words. These three-word descriptions provide a concise overview of how the users perceived the application.

They described it as easy to use.

The app's reliability was another frequently highlighted point. Users appreciated its consistency and predictability, noting that it sent regular reminders and functioned without any apparent glitches.

The app's usefulness was acknowledged in terms of its positive impact on monitoring users' health and collecting important research data.

The app was also described as reassuring, providing participants with a sense of security and support. Knowing they could report symptoms or adverse effects at any time and receive appropriate responses contributed to a more secure experience.

Finally, the app's effectiveness was emphasized, not only in terms of ease of use but also in terms of the speed and efficiency of its responses to user input.

> *Entrevue 4 : "Rassurante, eh, pertinente puis facilitante je dirais"*
>
> *Entrevue 5 : Facilité, Efficacité, Utilité : "Ben là, un qui est facile, c'est la facilité d'usage, facilité. Mais en fait je pourrais dire efficacité dans le sens où, ben c'est peut-être pas efficacité le mot, mais pour dire qu'il y a un retour, tu sais, s'il y a quelque chose qui fonctionne pas, le retour est rapide là, c'est comme t'as vraiment l'impression qu'il y a une utilité. Ça fait tu trois mots ça? (rires) "*
>
> *Entrevue 6 : "Accessibilité, rapidité puis détaillé […]"*
>
> *Entrevue 7 : Fiabilité, utilité, rassurant : "Trois mots. Eh mon dieu. Bon ok,* ***fiabilité.*** *Qu'est-ce que je pourrais dire? Eh, ouin* ***utile*** *parce que comme je vous disais, ça peut être utile pour ben des femmes. Et ça peut être* ***rassurant*** *aussi"*

8. Suggestions for improving the content and functionality of the application

Some participants expressed a need for more human contact: they suggested more frequent interactions with healthcare professionals, either through phone calls or written messages, to humanize the experience and reduce anxiety associated with treatment.

> *"Ce serait un contact, soit écrit, un petit mot écrit, ou par téléphone, sans que ce soit journalier ou hebdomadaire, au moins une fois dans le cycle." (Entrevue 2)*

Some find that filling out questionnaires daily is excessive, especially in the absence of new symptoms. A weekly frequency or adjustments based on changes in their condition are suggested.

> *"Ben, c'est sûr que moi, ça aurait été plus pertinent et plus adapté de l'avoir une fois par semaine. Fait que moi, ça permet d'avoir vraiment un moment où je fais une rétroaction de la semaine que j'ai eue, les symptômes que j'ai ou que j'ai pas, puis d'avoir comme une fil conducteur par rapport à ça versus tous les jours. Ça c'est un point." (Entrevue 6)*
>
> *"Eh, s'il y avait eu un endroit pour indiquer – Je n'ai pas d'effets secondaires ni d'effets indésirables, ceux qui étaient cités, les quatre dans le questionnaire quotidien, et que ça s'est poursuivi dans la durée là, peut-être que, à ce moment-là, le questionnaire hebdomadaire aurait été suffisant, c'est ça, parce que, mais, mais, si j'en avais eu, j'aurais peut-être été bien contente de le signaler, et peut-être que quelqu'un m'aurait appelée ou m'aurait écrit. Je pense*

que le quotidien c'est bien, mais moi c'est ça qui est arrivé, c'est que j'en n'ai pas vraiment eu" (Entrevue 2)

"Ben le journalier, je trouvais, à tous les jours, c'était comme toujours pareil, les quatre mêmes, tu sais, comme le fait justement qu'on pouvait pas, si on a de quoi qui nous chicote qui n'est peut-être pas grave, ou qui n'est pas dans les symptômes hebdomadaires ou quoi que ce soit, tu sais qui était journaliers qu'on aurait pu ajouter tu sais, pour le mentionner là, mais sinon, mais peut-être le fait que j'avais pas les symptômes qui étaient décrits là, à part une petite nausée, pour moi, c'était comme, je le remplissais parce que c'était pas long à remplir, ça c'était la bonne chose là. Mais mettons que ça aurait été tous les jours les quatre symptômes, puis avoir à répondre à chacun des symptômes avec des catégories un petit peu, un peu plus, un peu plus. Si ça avait été plus long, ça m'aurait, j'aurais trouvé ça un peu plate dans le sens où si ça s'applique pas, ben ça te tente pas de répondre à des questions qui s'appliquent pas à toi là tu sais. Mais là, le fait que c'était court, léger, puis si t'avais aucun de ces symptômes-là, t'as juste à pas répondre." (Entrevue 5)

"En fait, c'était une fois par jour, je recevais un courriel, donc je devais ouvrir le courriel quand j'avais des symptômes à noter, c'est ça sur une base journalière. Donc si je n'avais pas de symptômes, je faisais juste effacer le courriel et c'est tout, pendant les trois premiers mois puis après ça ben c'était terminé. Pour ma part, ça l'a été un soulagement quand ça l'a été terminé (rires), ah oui! Je trouvais ça très, très lourd de recevoir un courriel par jour par rapport à ça. Je comprends le principe, je comprends que c'est pas tout le monde non plus qui est habile avec l'informatique, donc d'avoir un courriel par jour, d'avoir un lien, je comprends que pour certaines c'est beaucoup plus pratique puis accessible, mais pour ma part, j'aurais pu juste avoir un lien dans mes favoris ou quelque chose comme ça, puis j'y aurais été seulement dans les journées où j'aurais eu des symptômes." (Entrevue 6)

More specifically, participants suggested various improvements that would allow for greater flexibility in use and personalization of the tool. This particularly concerned the application's functions and content. Participants suggested including an option to report the absence of adverse effects: this would allow them to confirm that everything is fine, thus contributing to their peace of mind. Other participants would have appreciated being able to report symptoms or effects not listed in the current questionnaire options, highlighting the need for an "Other" field for greater specificity. Another participant suggested being able to detail or specify the symptom, in addition to having more explanation or a definition to refer to in order to assess the symptom's intensity.

"Eh, s'il y avait eu un endroit pour indiquer – Je n'ai pas d'effets secondaires ni d'effets indésirables [...] peut-être que, à ce moment-là, le questionnaire hebdomadaire aurait été suffisant." (Entrevue 2)

"Ah, non, moi j'aurais rajouté, comme je l'ai déjà signalé, une case pour dire – Pas d'effets secondaires, aucun effet secondaire [...] En plus, juste le fait que ce soit sur le questionnaire, on se dit – Ah c'est possible que j'en n'ai pas." (Entrevue 2)

"Ben l'outil était bien. C'est sûr que tu sais, quand ils m'ont parlé d'une application au départ, je m'attendais à ce que ce soit plus, plus applicatif et non pas dans un courriel, mais tu sais, l'outil en tant que tel allait bien. La seule chose [...] je trouvais que des fois, il y avait pas Autres symptômes, il l'avait à la semaine, mais il l'avait pas à la journée, fait que si jamais, on

dirait que c'était comme les symptômes les plus importants mais tu sais, des fois, j'aurais aimé ça avoir une option pour dire – Il m'est arrivé ça aujourd'hui, fait que, au quotidien, je trouvais que les questions étaient trop restrictives [...], c'est ça, je le disais quand ils m'appelaient mais aussi disons j'avais juste les autres symptômes, ben là j'osais pas appeler, fait que j'aurais peut-être aimé ça avoir un Autre, à la limite, eux autres, ils décideront s'ils font quelque chose avec ça ou pas mais, tu sais," (Entrevue 4)

"J'aurais aimé qu'il y aurait eu, en fait, pour le programme là, mais c'était bien, mais moi j'aurais aimé qu'on aurait pu comme un petit peu élaborer, parce que c'est difficile de dire beaucoup, pas beaucoup, c'est quoi beaucoup? C'est quoi pas beaucoup? tu sais (petit rire), c'est des symptômes qu'on connaît pas qui sont nouveaux pour nous, fait que c'est difficile d'évaluer tu sais, dans une échelle de 1 à 5, ou léger, mais ça vous empêche pas de manger, moyen, mais vous arrivez quand même à manger. Mais c'est comme difficile d'évaluer là tu sais, ça c'est pas tant pour les nausées mais pour les autres. Tu sais, comme il y avait un rash, mais pour moi un rash c'est que tu t'arraches la peau là (petit rire). L'explication de ce qui était un rash, je l'ai su après quand j'ai eu terminé après les 90 jours là, parce qu'on a arrêté deux semaines le ribociclib, et quand j'ai repris, j'ai fait une réaction allergique. Alors là, j'ai su c'était quoi un rash (petit rire) fait que, j'aurais aimé pouvoir dire – Ben c'est pas un rash, mais je sens ma peau sensible que, tu sais des choses comme ça là que j'aurais aimé pouvoir dire là tu sais, qui donnait pas la possibilité dans le programme" (Entrevue 5)

"Le sondage, je vais te dire une affaire, une fois que t'as répondu là, puis que tu pèses sur Enter, c'est fini, tu peux pas revenir, tu comprends? Tu fais ça à 3h du matin, c'est pas vrai, tu peux le faire avec un email, à 3h du matin, tu feel pas, tu peux le faire très tôt demain matin [...] Fait que c'est ça, mais c'est ça, ce que je pourrais reprocher, ce serait que, une fois que t'as pesé sur Enter c'est fini là, tu peux pas revenir dans le sondage puis changer les réponses ou ajouter ou tu sais [...]" (Entrevue 1)

According to some participants, weekly or bi-weekly questionnaires would be better received than daily ones, especially after the initial treatment phase. One participant suggested adding the option to indicate the need to be contacted by the treatment team based on the progress of their treatment. Finally, another participant suggested that previously submitted questionnaire responses could be adjusted based on the evolution or appearance of symptoms within the same day.

"Ben c'est sûr que moi, ça aurait été plus pertinent et plus adapté de l'avoir une fois par semaine." (Entrevue 6)

"D'ailleurs trois mois, je trouve que, d'après moi là, à moins que la personne soit très insécure mais trois mois, je trouve que c'est suffisant pour atteindre un niveau de confiance." (Entrevue 3)

"Fait que le 14e jour, au début, c'est sûr qu'on a besoin de leur parler mais si le 14e jour, on sait qu'on tombe en pause puis on sait que notre douleur va être contrôlable, on aurait peut-être, moi j'aimerais avoir la possibilité, j'aurais aimé avoir la possibilité de dire – Ben, vous avez pas besoin de me contacter, je suis à la fin de ma dose, puis je vais savoir, je sais quoi faire parce que c'est ça qui est merveilleux, c'est qu'avec le temps, on prend comme une habileté à connaitre nos symptômes, puis on se sent plus en confiance, on a le sentiment qu'on a un peu le contrôle de la situation là je peux dire" (Entrevue 3)

"Le sondage, je vais te dire une affaire, une fois que t'as répondu là, puis que tu pèses sur Enter, c'est fini, tu peux pas revenir, tu comprends? […] mais c'est ça, ce que je pourrais reprocher, ce serait que, une fois que t'as pesé sur Enter c'est fini là, tu peux pas revenir dans le sondage puis changer les réponses ou ajouter ou tu sais […] Moi le sondage, je vois ça simplement comme faire des statistiques" (Entrevue 1)

9. Recommendation of the app to other women

Most participants expressed a general recommendation to use the app with other women undergoing similar treatments, highlighting several reasons that vary depending on specific circumstances and individual needs for support, managing side effects, and emergency assistance.

First, they valued the app for the sense of ongoing support it provides. They emphasized that, despite the physical distance, the app creates a connection with the healthcare team, which is reassuring and reduces the stress associated with treatment. Testimonials suggest that the app is particularly useful at the beginning of treatment, when the unpredictability of side effects can be daunting and stressful. The app helps manage these effects and offers practical advice for mitigating them. Some participants appreciated having a way to communicate changes in side effects and receive appropriate responses from the medical team. This could be especially helpful for some women who may be hesitant to initiate communication with their healthcare team. The app offers a less intimidating way to report problems and receive support.

Furthermore, one participant sees the app as a crucial tool in emergencies, enabling women to communicate quickly and effectively with the medical team, which can be essential in cases of severe or unexpected reactions to treatments.

Interviewer *: "Puis pour quelle raison vous pensez que ça pourrait être bénéfique pour d'autres femmes?" Participante (P) : "(silence) Parce que c'est quand même un suivi. On se sent quand même liée même si c'est tenu comme fil (petit rire)"* (Entrevue 2)

"Ben pour l'instabilité des effets secondaires, surtout au début, le temps de se connaitre, ça peut être stressant de passer des grosses journées à pas trop loin de la toilette là." (Entrevue 4)

Interviewer *: "Est-ce que vous recommanderiez d'ajouter ce type d'outil-là aux soins, aux services qui sont déjà offerts aux femmes qui ont votre traitement? "* Participante *: "[…] je pense que c'est bon pour vous (à propos de l'équipe de recherche, puis c'est bon pour elle(à propos d'une autre femme qui a le même type de traitement) […] Ben parce que comme je disais tantôt, c'est sûr que si t'es très sensible puis que là, ça te rappelle à tous les jours que t'as le cancer, mais si c'est pas ça, c'est d'autre chose qui va te le rappeler (petit rire). Mais, dans le sens où c'est pas tout le monde qui va aller mettons aux renseignements sur tel effet, sur tel effet, tu sais, puis de la recherche, on s'entend que si tu vas chercher sur internet, on te montre toujours tous les pires cas, fait que ça s'adresse pas nécessairement à toi, c'est pas bon tu sais. Fait que là, en ayant cet outil-là, ben si la personne, elle a une inquiétude, n'importe*

quoi, qu'elle y va sur le site puis qu'elle le complète, puis qu'il y a un retour de ça, ben c'est rassurant pour elle. " (Entrevue 5)

> Interviewer *: "Est-ce que vous recommanderiez d'utiliser cet outil-là à d'autres femmes qui ont un traitement comme vous?"* Participante *: "Oui, oui, parce qu'on est comme toutes différentes, on a des besoins différents puis je vous avouerais que tu sais, quand on subit ces traitements-là, des fois on est comme un peu sur le radar aussi puis selon l'acceptation du début de la maladie, tu sais, je veux dire, on n'a pas le même niveau d'énergie, il y en a qui ont vraiment besoin de se rassurer à tous les jours, qu'est-ce qu'il en est, de se positionner, de se rappeler les symptômes, de se rappeler les interventions, […]. Mais il y en a qui sont moins confiantes, ils sont moins sûres d'elles puis elles ont besoin d'être accompagnées au niveau, qui ont moins d'autonomie. Mais moi, ce n'était pas le cas là. Mais je comprends entièrement les besoins pour certaines femmes à ce niveau-là"* (Entrevue 6)

> *"Aider pour leur traitement, aider pour leur traitement, moi je pense que certaines femmes qui, je connais pas tout le monde là, moi quand il y a de quoi, ça me gêne pas d'avancer puis de demander tu comprends, mais peut-être qu'il y a des femmes qui sont plus, ce sondage-là peut aider certaines femmes qui sont plus gênées d'appeler."* (Entrevue 1)

> *"Oui parce que ça peut être utile en cas d'urgence, parce que à ce moment-là, si t'as de quoi, ils vont t'appeler tout de suite grâce au sondage."* (Entrevue 1)

10. Expanded Use of the Application

10.1 Use of the Application Beyond Targeted Treatment

Participants suggested that the application could be used for a wider range of medications and treatments, making it useful far beyond its initial purpose. This adaptability would allow for the management of diverse medical conditions and their associated treatments. One participant also saw added value in the application within a broader healthcare context, particularly for monitoring hospital chemotherapy treatments once patients are at home.

> *"Moi je vous dirais, s'il y a d'autres médicaments qui ont des effets secondaires importants, je pense que ça serait bénéfique là […] Ben je vous dirais que oui, puis tu sais, vous me faites penser quand j'étais en traitement de chimiothérapie, pendant que, dans le fond, la première chimio que j'ai eue, c'est peut-être des affaires qui peuvent être pertinentes aussi là, de savoir les effets secondaires qui sont après les traitements…"* (Entrevue 3)

10.2 Reducing the burden on the healthcare system

According to some participants, the application could reduce the frequency of calls to healthcare professionals and allow for more structured communication based on actual data about symptoms experienced day after day.

> *"Ben, rien à dire au niveau des services qu'on me donne. Tu sais, moi j'ai été super bien servie, écoute, le monde était exceptionnel, tout le monde était exceptionnel, jusqu'en parlant avec*

vous, je me dis – Peut-être que si j'avais eu ça peut-être que j'aurais peut-être moins appelé des fois, soit les infirmières pivots pour avoir des questions, parce qu'il y a des affaires qui m'inquiétaient, ou tu sais, là j'aurais su que dans le fond je complétais mon formulaire, puis avec mes réponses, si ça nécessitait un appel, il y a quelqu'un qui m'aurait appelée." (Entrevue 4)

"S'il y en aurait une, ça l'aurait été, je vous avouerais que quand je vais rencontrer mon oncologue, qu'elle l'ait, qu'elle puisse dire – Tu me dis que ça bien été, après on va aller voir objectivement dans ton questionnaire, on aurait eu un fil conducteur de mes symptômes et non moi qui les rapporte comme ça, sans dire de date, sans avoir des éléments précis, le questionnaire le permettait." (Entrevue 6)

10.3 Long-term use of the application

Participants' opinions regarding the potential of using the application for long-term patient monitoring were mixed. One participant felt it would allow for monitoring the evolution of symptoms (adverse effects) after initial stabilization.

"D'ailleurs trois mois, je trouve que, d'après moi là, à moins que la personne soit très insécure mais trois mois, je trouve que c'est suffisant pour atteindre un niveau de confiance. Comme là, moi je me sens, c'est sûr que si jamais j'avais un problème, je sais que je peux appeler à la pharmacie mais j'ai pu besoin de compléter un questionnaire à tous les jours pour dire comment ça se passe, parce que là, après 3 mois, je trouve que c'est un bon délai pour prendre notre autonomie puis être confiante avec nos symptômes" (Entrevue 1)

"Ben ce que je dirais, à long terme non, je pense que c'est plus en début de traitement. Si j'avais à le remplir aujourd'hui, j'ai quand même des suivis réguliers avec le médecin, tu sais, j'ai pu de surprise, je commence à connaitre un peu mes affaires, je suis pas sûre que tu sais, en date d'aujourd'hui, à part si c'était une fois par mois pour des fins de, ben je dis statistiques mais de compréhension du médicament, je sais pas si tout le monde le ferait, moi ça me dérangerait pas de le faire mais tu sais, je pense que tu sais, pour le suivi comme au début du médicament, je suis pas sûre que ce serait encore pertinent." (Entrevue 4)

"Non, non. Ce que le questionnaire permettait en fait, c'est que lui, le questionnaire me laisse des traces à tous les jours quand on a des symptômes, donc une fois qu'on rencontre notre oncologue..." (Entrevue 6).

10.4 Improving the quality of care through proactive interventions

According to the participants, the use of the application would allow for proactive intervention by healthcare professionals, thanks to the real-time reception of data on patients' symptoms, thus facilitating faster and more targeted responses.

"Moi c'est vraiment le fait que quand t'es malade à la maison, si tu remplis un formulaire le matin de ce qui s'est passé, tu sais, tu dis – Le professionnel qui va lire mon rapport, s'il pense

que je dois avoir un suivi puis qu'il y a quelque chose qui n'est pas correct, il va m'appeler..." (Entrevue 4)

"Parfait, puis eux ça faite, si ça avait été quelqu'un, je me mets dans la peau de quelqu'un qui est plus insécure, qui est plus que moi puis que ça lui dérange pas de déranger, ben peut-être que ça aurait fait beaucoup d'appels sur les répondeurs des pharmaciens." (Entrevue 4)

"Ben je vous dirais que oui, puis tu sais, vous me faites penser quand j'étais en traitement de chimiothérapie, pendant que, dans le fond, la première chimio que j'ai eue, c'est peut-être des affaires qui peuvent être pertinentes aussi là, de savoir les effets secondaires qui sont après les traitements, est-ce que quelqu'un est encore malade? Est-ce que, parce que moi je sais qu'à mon deuxième protocole, a fallu que j'appelle des pharmaciens puis ces affaires-là parce que j'ai attendu longtemps avant d'appeler, fait que je me suis ramassée plus maganée." (Entrevue 5)
