# Supplementary material for: Development and Evaluation of a Web-Based App for Adverse Effect Management in Breast Cancer Patients Treated with Oral Targeted Therapy or Chemotherapy: Findings from a Pilot Study
Source: Curr Oncol. 2026 May 7;33(5):272. doi: 10.3390/curroncol33050272 (PMC13205787; doi:10.3390/curroncol33050272)
Supplement: Supplementary file 1 [file curroncol-33-00272-s001.zip › File S4 JCH.pdf]

**SUPPLEMENTARY MATERIALS**

**QUOTES FROM THE SEMI-STRUCTURED INTERVIEWS WITH HEALTH PROFESSIONALS**

**Table.** Quotes from the healthcare professionals

| Theme | Subtheme | Quotes |
|---|---|---|
| Perceived impact on practice | 1.1 Directing consultations | 1.1 *"Hum, ça été une bonne expérience en ce qui me concerne, je l'utilisais [à propos du rapport des effets indésirables mis au dossier de la patiente] comme un guide de départ pour questionner mes patientes sur justement les effets secondaires de leur thérapie endocrinienne. Fait que par exemple, si je voyais que la patiente était plein de bouffées de chaleur, ben ça me guidait pour commencer l'entrevue avec la patiente" (Entrevue 4)* |
| | | Hum, it has been a good experience as far as I am concerned, I used it [about the report of adverse effects added to the patient's file] as a starting guide to actually question my patients about the side effects of their endocrine therapy. So for example, if I say that the patient had a lot of hot flashes, well then it would guide me on how to start the patient's interview. (Interview 4) |
| | 1.2 Raising awareness among patients | 1.2 *" [...] ça les conscientisait à leurs effets indésirables finalement pour pas laisser ça sous silence." (Entrevue 3)* |
| | | It helping them getting aware of their side effects so they would not ignored them. (Interview 3) |
| | 1.3 More frequent communication | 1.3 *"Moi j'ai l'impression que, il y a des patientes qui nous auraient pas appelé, tu sais, qui avaient des effets secondaires de grade 2 mais que si ça avait pas été de l'appli, je suis pas sûre qu'ils nous auraient appelé puis qu'elles auraient eu cet effet secondaire-là à la maison, je sais pas comment elles l'auraient géré. Je pense que ça généré plus de contacts avec les patientes, avec le projet appli, qu'en temps normal, quand on rencontre les patientes quand elles débutent un nouveau traitement. Ensuite on leur remet la documentation puis les trucs, puis qu'on leur dit – « Ben vous nous appellerez si ça va pas ». Ben je pense que ces patientes-là, soit qu'elles se gèrent toute seules avec les documents qu'on a donnés, ou qui se gèrent mal puis qui nous appellent pas puis ça va mal, ça je le sais pas, mais je pense qu'on a eu plus d'interactions avec les patientes par* |

| Theme | Subtheme | Quotes |
|---|---|---|
| | | *les alertes de l'appli qu'on en aurait eu en temps normal." (Entrevue 5)* |
| | | Me, I believe there are some patient who wouldn't have called us, you know, who were having grade 2 side effects, but it it hadn't been for the app, I am not sure they would have called and they would have this side effects at home, I don't know how they would have handled it. I think it has generated more contacts with the patients, with the app project, then normally, when we meet the patients when they start a new treatment. Then we provide them with documentations and stuff, and we tell them – "Well you can call if something's wrong". Well I think these patients, either they manage on their own with the documents we've given them, or they don't manage well and don't call us and then things go bad, that I don't know, but I think we've had more interactions with the patients with the app's alerts than we would have had under circumstances. (Interview 5) |
| | 1.4 Confusion regarding the follow-up | 1.4 *" […] c'est quand on avait le message d'alerte qui popait, si on veut, dans notre adresse, notre boîte courriel, puis là, à ce moment-là, on se disait – Oh ok, oh, on est en alerte, il y a un message – Ok, ben là c'est vrai, faut attendre, en principe, la patiente est supposée de nous appeler. Fait que là,elle, elle nous appelle pas tout de suite. Ok, là on attend-tu? C'est quand il faut l'appeler? Si elle nous appelle pas, est-ce qu'on va l'appeler cet après-midi? Fait que tu sais, on n'est pas du genre à laisser un suivi, comme quelqu'un qui nous laisse un message sur notre boîte vocale, on n'appelle pas dans les 48h, tu sais, on appelle dès que, on les prend au fur et à mesure parce qu'on sait pas combien qu'il va y en avoir d'autres plus tard dans la journée puis qu'on veut être capable de répondre à tout le monde. Fait que ça nous amenait un peu cette ambivalence, - Ok, ben là, est-ce qu'on appelle? On n'appelle pas? Ok là on attendait, on était un petit peu stand by puis là, on finissait par appeler la patiente dans l'après-midi si jamais elle nous avait pas appelés. Il y avait un petit peu cette petite incertitude-là à ce moment-là, quand on avait la notification. Puis tu sais, vu qu'on était en mode vigilance quand on recevait ces messages-là, on était comme tout énervés (rires). Fait que ça l'a amené un peu cette gestion-là de nous-même finalement, d'être capable de se gérer* |

| Theme | Subtheme | Quotes |
|---|---|---|
| | | *d'avoir une notification, sans savoir si c'était grave ou non".  (Entrevue 3)* |
| | | […] it was when we got the alert message popping up, if you want, in our inbox, our email, and that we'd be like "Oh okay, right, we are on alert, there's a message". – Ok, then, right, we need to wait, in principle, the patient is supposed to call us. So then, she does not call us right away. Ok, then, now we wait? When are we supposed to call? If she doesn't call , are we supposed to call this afternoon? So, you know, we are not the type to just leave a follow-up hanging, like someone who leave a message on a voicemail and we do not call back for 48hours, you know, we call back as soon as, we take them as they come because we do not know how many other there will be later in the day and we wanna be able to call everyone back.<br>So it brought up a kind of ambivalence, like – Ok so do we call? Do we not call? Ok we'd be waiting a bit, being a bit on standby then we'd end up calling the patient in the afternoon if she ended up not calling back. There was a bit of uncertainty at that time, when we got a notification. Then you know, as we were in vigilance mode, when we received these messages, we were kinda agitated (laugh). So it ended up leading to a bit of self-management, being able to deal with getting a notification without knowing whether or not it was serious. (Interview 3) |
| Implementation in everyday practice | 2.1 Example of a facilitating factor | 2.1 *"[...] avant de partir quelque chose comme ça, il faut vraiment se parler en équipe puis travailler tout le monde ensemble. Il n'y a pas juste les infirmières pivots, il y a autant les pharmaciens puis tu sais, au-delà de ça, il y a les équipes psychosociales qui sont là-dedans, qui doivent accompagner les patientes qui sont en détresse, qui sont anxieuses, ben de savoir c'est quoi, je pense que c'est important aussi pour ces personnes-là, puis l'équipe médicale, tout le monde, tu sais, c'est pas juste un silo, c'est plusieurs personnes qui doivent toutes travailler ensemble" (Entrevue 2)* |
| | | […] before starting something like that, it must really be talked through as a team then working all together. It is not just the nurses navigator, there are the pharmacists also then, you know, beyond that, there are the psychosocial teams who are part of it, who must support the patients who are distressed and anxious, like knowing what it is, I think it is also |

| Theme | Subtheme | Quotes |
|---|---|---|
| | | important for these people then the medical team, everyone, you know, not just one silo, it's several people who must work together. (Interview 2) |
| | 2.2 Example of a barrier | 2.2 *"Puis il y a aussi d'expliquer aux patientes comment ça va fonctionner. Fait que ça, c'est sûr que c'est du temps aussi, fait que ça c'est un peu une barrière je dirais. Si quelqu'un leur expliquait puis qu'elles pouvaient s'inscrire toutes seules, puis qu'on aurait juste à gérer les alertes, moi je verrais ça très facile" (Entrevue 5)* |
| | | Then there is also to explanation to be given to the patients as to how it will work. Then that must take some time also, so that is another kind of barrier I would say. If someone provide them with the explanations then they could enroll themselves, then we would only have to manage the alerts, me I would find it very easy. (Interview 5) |

## RESULTS OF SEMI-STRUCTURED INTERVIEWS WITH HEALTH PROFESSIONALS

### 1. Summary of the Approach

A total of five interviews were conducted in the fall of 2023 with healthcare professionals from the Breast Disease Centre (BDC), including pharmacists, oncology nurse navigators, and physician oncologists. These professionals were identified by the research team (J-C Hogues). The interviews were conducted by telephone (Julie Lapointe) using the interview guide provided in the appendix. The interviews were transcribed in full. A thematic analysis, assisted by NVivo software, was performed by a research professional (A. Baghdadli) in collaboration with S. Lauzier. This report presents the main findings of these interviews. For each identified theme and sub-theme, several excerpts from the interviews are provided. The most relevant excerpts may be selected for publication.

### 2. Experience Using Adverse Event Reports

Three participants mentioned challenges in accessing adverse event reports, particularly in the context where they were only available to study participants.

The first challenge mentioned was identifying the patients for whom the report was available. The second challenge was finding the adverse event reports located in the patient's external medical record. This meant having to navigate through the records more extensively to locate the document, which is neither intuitive nor efficient in routine practice.

One of these participants noted that when they consulted the reports, it was by chance while searching through the patient's medical record for other information. This same participant explained that they only consulted them when patients mentioned the report.

Finally, another professional mentioned not having seen or consulted the reports because they found the information they were looking for in the team's follow-up notes.

> Interviewer *: "[...] avez-vous vu et consulté dans le dossier des rapports des effets secondaires qui provenaient de l'application?"* Participant : *"Oui [...] En fait, ben il y avait différents enjeux. Le premier enjeu, c'était de savoir que la patiente était dans l'étude. Fait que moi, j'essayais de m'écrire une petite note dans mes notes. J'écrivais Opal parce qu'il faut savoir qu'avant, le projet devait être fait avec l'application Opal. Des fois, j'écrivais Opal dans mes notes ou j'écrivais participe projet Appli, fait que comme ça, ben ça me faisait y penser. Puis là, ben en fait, ce qui était compliqué, c'est qu'il fallait aller dans Résumé extérieur pour voir le fichier PDF qui avait été scanné[...] mais quand je notais que c'était une patiente qui participait au projet, j'essayais d'aller trouver l'information dans le DPE"* (Entrevue 1)

> Interviewer *: "avez-vous vu dans les dossiers de vos patientes des rapports d'effets secondaires provenant de l'application?"* Participant : *"Oui, moi je les voyais parce que je savais où qu'ils*

*étaient, ils étaient dans une page différente. Mais oui, effectivement, je pouvais les voir [...] Ben dans le fond, l'utilisation, moi j'ai vu les rapports mais on s'en servait pas, tu sais, en fait on savait pas les patientes qui étaient dedans, excepté moi quelques patientes qui me l'ont mentionné, mais sinon, moi puis mes collègues, on savait pas quelles patientes qui avaient été sélectionnées pour faire partie de ce projet-là. Donc systématiquement on n'allait pas voir dans les dossiers c'était quoi, il fallait fouiller pour le trouver là.* Interviewer*: "Fait que quand vous les avez vus, c'était fortuitement ceux que vous aviez vus, c'était parce que les clients vous le disaient ou fortuitement là, au cours de, quand vous regardiez le dossier"* Participant *: "Fortuitement parce que des fois, on avait, tu sais, une patiente appelait pour une autre raison, puis tu sais, souvent on va fouiller dans le dossier, puis moi je tombais dessus là, comme ça là"* (Entrevue 2)

Interviewer *: "[...] avez-vous vu dans le dossier de vos patientes des rapports d'effets secondaires provenant de l'application?* Participant *: "Dans les dossiers des patientes, j'avoue, moi j'en n'ai pas, vous voulez dire comme les rapports han, non, j'avoue, moi je les ai pas vu les rapports. J'ai vu nos suivis à nous qu'on a faits en lien avec ceux-là, mais j'ai pas eu besoin d'aller réouvrir ces dossiers-là par après. Fait que donc c'est vrai, j'ai pas vu les rapports qui ont été déposés au dossier. Vous me rappelez ça en même temps, oui"* (Entrevue 3)

When the reports were reviewed, three participants described them as easy to read and understand.

Interviewer*: "Est-ce que vous trouvez que les rapports étaient faciles à lire, à consulter? C'était bien fait, ça vous donnait l'information que vous aviez besoin?"* Participant *: "Absolument"* (Entrevue 4)

Interviewer*: "Quand vous le regardiez ce rapport-là, le trouviez-vous facile à comprendre?"* Participant *: "Ben moi oui, parce que tu sais, j'ai quand même fait partie du processus. Ça fait que je savais un peu, j'ai travaillé avec [3 noms retirés], ça fait que oui, je voyais. Mais même si j'avais pas été dedans, oui, j'aurais compris là, c'est quelque chose qui est facile pour moi"* (Entrevue 2)

*"[...] c'était quand même facile à lire (à propos des rapports d'effets indésirables), c'était assez aéré là[...]"* (Entrevue 1)

One participant was less than impressed with the layout/aesthetics of the digitized reports in the patients' medical records. He suggested that reports including color-coded progress graphs would have been more appreciated.

*"[...] c'est sûr que le visuel était pas super, mais quand je notais que c'était une patiente qui participait au projet, j'essayais d'aller trouver l'information dans le DPE"* (Entrevue 1)

*"[...] C'est juste que c'était pas des rapports, par exemple, il n'y avait pas de graphique. Ça aurait été intéressant de voir des graphiques qui comparent d'une semaine à l'autre l'évolution des symptômes. On aurait pu avoir des codes couleurs puis tout ça, mais bon, malheureusement*

*c'était juste la question avec le score qui était écrit en lettres ou en chiffres donc, mais bon, ça on le savait quand on a commencé le projet".* (Entrevue 1)

Other healthcare professionals consulted for this study expressed some reservations about the usefulness of reviewing and using adverse event reports in patients' medical records. This was primarily due to the time it took for these documents to be added to the records (2-3 weeks, according to one participant). Two participants explained that although they had reviewed the adverse event reports, they did not find them useful for various reasons. Either the information in the reports did not require their intervention, or they had already taken action before receiving the document, making reporting adverse events redundant and unnecessary, unlike email alerts, which were received quickly and allowed for rapid and effective intervention.

*"Des fois c'est parce que, aussi, c'était un problème qui était connu depuis les derniers jours, donc la patiente, elle savait déjà un peu quoi faire, mais tu sais, elle savait, oui, ça l'avait augmenté, mais c'était pas nécessairement plus élevé que la dernière fois qu'on s'était parlés, il y avait ça aussi là tu sais. Fait que quand on était la même personne qui recevait le même message, et que la personne savait que c'était la même personne, le même pharmacien qui recevait le message, il disait – Ah ben oui, on a déjà un suivi qui est introduit, qui est inséré depuis la dernière semaine, qu'on est déjà au courant. Fait que dans ce temps-là, on sait au moins que le drapeau a été levé. Donc on savait qu'on avait déjà des suivis qui étaient prévus finalement dans ce temps-là"* (Entrevue 3)

*"Moi je m'en suis pas vraiment servi (à propos des rapports d'effets indésirables), je les ai vus mais je m'en suis pas servi, parce que en tout cas, j'en ai vus pas tant que ça, mais ceux que j'ai vus, c'était des effets secondaires de grade 1 ou c'était des effets secondaires qui étaient plus graves, puis à ce moment-là, j'avais déjà parlé avec la patiente. Fait que moi ça été plus mettons, j'ai constaté quelque chose que je savais déjà ou quelque chose qui n'avait pas d'impact pour moi, qui m'amenait pas à porter une action"* (Entrevue 5)

*"Nous autres, on était avisés par courriel quand les patientes avaient un effet secondaire de grade 2 ou plus. Fait qu'on recevait un courriel, fait que ce n'était pas nécessairement un, c'était pas un rapport, c'était un courriel. Mais je sais qu'il y avait aussi les rapports papier qui étaient versés au DPE à toutes les semaines. Fait que je pensais que c'était ces rapports-là versés au DPE à toutes les semaines qu'on parlait, une fois par semaine. Mais moi, quand ils étaient versés au DPE, ben soit que je n'avais pas entendu parler, puis c'était correct que je n'avais pas entendu parler, puis j'avais rien à faire, ou j'avais déjà reçu un courriel précédemment, puis j'étais déjà au courant. Fait que pour moi, ça c'était pas une information que j'utilisais parce que moi, ce qui était important pour moi, ben ce qu'on utilisait, c'était vraiment le courriel qu'on recevait. Le courriel d'alerte comme tel pour dire qu'une patiente avait des effets secondaires de grade 2 ou plus, donc plus graves, fait que là, c'était là-dessus qu'on agissait"* (Entrevue 5)

*"[...] Mais c'est sûr qu'un des problèmes (concernant l'accès au rapport d'effets indésriables) c'est que c'était pas à jour, fait que même si [nom retiré] envoyait à chaque semaine des formulaires, ils étaient pas nécessairement scannés rapidement. Fait que des fois, on voyait la*

*patiente, puis il pouvait y avoir 2-3 semaines qui s'étaient écoulées entre le moment où elle avait complété un rapport, puis le moment où il était mis au dossier. Fait que c'est sûr que ça faisait que c'était un petit peu moins utile là [...]"* (Entrevue 1)

*"Les pharmaciens, nous autres, on recevait les courriels, mais les autres professionnels ne recevaient pas de courriel, ils voyaient les rapports au DPE, puis les notes aussi qu'on écrivait, parce que quand on avait un courriel d'une patiente, ben on écrivait toujours une note par la suite au DPE pour dire – Ben on a reçu un courriel d'alerte de l'appli, on a appelé la patiente, telle, telle, telle chose, elle a bien fait, elle a bien suivi les recommandations ou pas. Fait que les professionnels pouvaient aussi voir en temps réel la note qu'on avait faite au DPE en plus du rapport"* (Entrevue 5)

## 3. Perceived Impact of the Application

### 3.1 Improvement of Practice

Regarding the perceived impact of the application on practice and the management of adverse events, two participants stated that adverse event reports were useful for initiating and guiding discussions with patients during the assessment of adverse events. One participant explained that despite the delays in filing the adverse event report, having it added value to their practice, "a plus." They were able to use it to initiate and guide their discussions with patients, and it allowed for a faster and more effective assessment of adverse events and made interventions more targeted.

*"Hum, ça été une bonne expérience en ce qui me concerne, je l'utilisais (à propos du rapport des effets indésirables mis au dossier de la patiente) comme un guide de départ pour questionner mes patientes sur justement les effets secondaires de leur thérapie endocrinienne. Fait que par exemple, si je voyais que la patiente était plein de bouffées de chaleur, ben ça me guidait pour commencer l'entrevue avec la patiente"* (Entrevue 4)

*"[...] Peut-être que si ça avait été en temps réel (à propos de la disponibilité des rapports d'effets indésirables), peut-être plus, mais là, comme c'était pas en temps réel, je trouvais ça intéressant parce que ça ouvrait la discussion puis ça permettait de pas poser de questions inutiles. Mais en même temps, comme il était pas en temps réel, ben fallait quand même des fois que tu reposes des questions parce que c'était pas en temps réel puis que tu voulais pas rien manquer. Fait que je trouvais ça un plus, mais c'est pas parfait encore pour utilisation au quotidien je dirais"* (Entrevue 1)

*"[...] Ben en fait, l'utilisation que j'en faisais (à propos des rapports d'effets indésirables) c'est qu'en début d'entrevue, quand je voyais qu'on avait un rapport, ben je disais – Bon madame, tu sais, j'ai vu vos effets secondaires, vous avez eu par exemple pas de nausées mais vous avez eu de la diarrhée, fait que je l'utilisais un peu pour commencer la conversation puis le questionnaire avec la patiente. Mais c'est sûr qu'un des problèmes (concernant l'accès au rapport d'effets indésirables) c'est que c'était pas à jour, [...] Fait que des fois, on voyait la patiente, puis il pouvait y avoir 2-3 semaines qui s'étaient écoulées entre le moment où elle avait complété un rapport, puis le moment où il était mis au dossier. Fait que c'est sûr que ça*

*faisait que c'était un petit peu moins utile là, mais je m'en servais, c'est ça, pour un peu commencer l'entrevue, puis en me disant – Ben si la patiente a pas eu ces effets secondaires-là, ben je vais pas perdre du temps à lui poser la question, tu sais j'irai pas lui poser – Madame, avez-vous eu mal au cœur? Si dans le fond c'est écrit qu'elle a pas eu mal au cœur, fait que ça sauve un peu du temps, puis ça permet de focusser sur d'autres points peut-être plus significatifs pour les patientes […]* (Entrevue 1)

According to one healthcare professional, using this app and accessing the adverse event report has improved their practice. They explain that it's a kind of adverse event screening tool that allows patients to feel more comfortable discussing certain symptoms. They believe that patients feel validated in their experience of adverse effects and might not have discussed these symptoms if they hadn't been prompted to answer the app's questions due to embarrassment or forgetfulness. Similarly, another participant explains that the app has improved their practice by allowing patients to reflect on their symptoms and become aware of their existence and evolution.

> *"Oui. Parce que je pense que les patientes, vu qu'il y a comme un outil de dépistage on va dire, se sentent plus à l'aise de le mentionner puis d'en parler après ça avec nous. Alors que quand mettons les patientes viennent puis elles n'ont pas eu d'outil comme ça, ben peut-être qu'elles sont gênées de mentionner les effets secondaires, fait qu'elles ne veulent pas en parler ou elles oublient d'en parler. Puis nous, ben des fois on est pressés, fait qu'on n'aborde pas tous les effets secondaires, comme par exemple la sécheresse vaginale, des fois on passe vite par-dessus parce que nous on est pressés, les patientes sont gênées, fait que là, ben ça fait en sorte que personne en parle. Tandis que si elles ont eu à le cocher ou elles l'ont vu sur une feuille de papier, ben à ce moment-là, elles sont comme – Ah oui c'est vrai, faut que je parle de ça. Puis c'est valide mon symptôme parce que je l'ai lu et on m'a questionnée là-dessus"* (Entrevue 4)
>
> Interviewer*: "Est-ce que vous trouvez qu'en général, l'application, les rapports des effets secondaires auto-rapportés, ça l'améliorait le suivi de vos patientes?"* Participant : *"Ben moi je pense que oui, parce que ça les conscientisait à leurs effets indésirables finalement pour pas laisser ça sous silence. Mais sinon, c'est ça, je pense que c'est un outil qui est, un outil technologique c'est sûr que c'est pas fait pour tout le monde évidemment là, mais je pense que ça vaut, tous les outils possibles permettent que les intéressés, les patients aux traitements puis d'être conscientes de tout ça, je pense que ça va toujours être quelque chose qui va être facilitant là. Hum"* (Entrevue 3)

Similarly, one participant explained that, in his opinion, the app improved his practice by allowing for more proactive symptom management and encouraging more frequent interactions. He suggested that patients might not have initiated contact with the healthcare team as frequently as they did using the app and might have managed their symptoms less effectively on their own, relying solely on the information sheets provided by the pharmacy.

> Interviewer *: "est-ce que vous trouvez que ça l'a eu un impact sur votre prestation de services, sur les visites, les appels puis la gestion des services aux patientes?"* Participant : *"Moi j'ai l'impression que, il y a des patientes qui nous auraient pas appelé, tu sais, qui avaient des effets secondaires de grade 2 mais que si ça avait pas été de l'appli, je suis pas sûre qu'ils nous auraient appelé puis qu'elles auraient eu cet effet secondaire-là à la maison, je sais pas*

*comment est-ce qu'elles l'auraient géré. Je pense que ça généré plus de contacts avec les patientes, avec le projet appli, qu'en temps normal, quand il n'y a pas le projet appli, qu'on rencontre les patientes quand elles débutent un nouveau traitement. Ensuite on leur remet la documentation puis les trucs, puis qu'on leur dit – Ben vous nous appellerez si ça va pas. Ben je pense que ces patientes-là, soit qu'elles se gèrent toute seules avec les documents qu'on a donnés, ou qui se gèrent mal puis qui nous appellent pas puis ça va mal, ça je le sais pas, mais je pense qu'on a eu plus d'interactions avec les patientes par les alertes de l'appli qu'on en aurait eu en temps normal. C'est mon feeling"* (Entrevue 5)

Another participant also believes that the application played a role in improving his practice by making patients more aware of their symptoms.

> Interviewer *: "Est-ce que vous trouvez qu'en général, l'application, les rapports des effets secondaires auto-rapportés, ça l'améliorait le suivi de vos patientes?"* Participant *: "Ben moi je pense que oui, parce que ça les conscientisait à leurs effets indésirables finalement pour pas laisser ça sous silence. Ok, moi je pense que oui [...] fait que ça permet de mieux suivre encore, je pense, celles qui étaient déjà bien suivies je crois, parce que c'est des patientes quand même plus jeunes, puis elles étaient autonomes puis elles se gênaient pas de nous contacter"* (Entrevue 3)

One participant explained that the alerts received after patients reported adverse effects led to some uncertainty regarding initiating calls to follow up on reported symptoms. According to him, patients did not always call as prompted by the application, which added stress and led him to initiate calls to prevent the symptoms from worsening.

> *"Oui, fait que moi j'avoue, l'expérience que j'ai eue (à propos de l'expérience de l'utilisation des rapports des effets indésirables) principalement, c'est quand on avait le message d'alerte qui popait, si on veut, dans notre adresse, notre boîte courriel, puis là, à ce moment-là, on se disait – Oh ok, oh, on est en alerte, il y a un message, ok tout ça, là on se disait – Ok, ben là c'est vrai, faut attendre, en principe, la patiente est supposée de nous appeler. Fait que là, ok, bon là, elle, elle nous appelle pas tout de suite. Ok, là on attend-tu? C'est quand il faut l'appeler? Si elle nous appelle pas, est-ce qu'on va l'appeler cet après-midi? Fait que tu sais, on n'est pas du genre à laisser un suivi, comme quelqu'un qui nous laisse un message sur notre boîte vocale, on n'appelle pas dans les 48h, tu sais, on appelle dès que, on les prend au fur et à mesure parce qu'on sait pas combien qu'il va y en avoir d'autres plus tard dans la journée puis qu'on veut être capable de répondre à tout le monde, fait qu'on est plus dans ce style-là. Fait que ça nous amenait un peu cette ambivalence, - Ok, ben là, est-ce qu'on appelle? On n'appelle pas? Ok là on attendait, on était un petit peu stand by puis là, on finissait par appeler la patiente dans l'après-midi si jamais elle nous avait pas appelés. Il y avait un petit peu cette petite incertitude-là à ce moment-là, quand on avait la notification. Puis tu sais, vu qu'on était en mode vigilance quand on recevait ces messages-là, on était comme tout énervés (rires). Fait que ça l'a amené un peu cette gestion-là de nous-même finalement, d'être capable de se gérer d'avoir une notification, sans savoir si c'était grave ou non. J'ai l'impression que si ça avait été quelque chose de grave, la patiente nous aurait appelé directement là, elle n'aurait pas attendu. Mais j'avoue que j'ai trouvé qu'il y avait pas, les patientes appelaient pas tant que ça spontanément quand il y avait un message d'alerte* Interviewer *: "D'accord. Pensez-vous que*

*c'est parce que les patients ne réalisaient pas que ça générait un message d'alerte pour vous?"* Participant : *"Eh non, je pense qu'elles le savaient, je pense qu'elles savaient que ça envoyait un avis que, je pense que ça leur faisait un message qui leur disait – Veuillez contacter, puis me semble que ça leur disait qu'un message avait été envoyé. Je pense que c'est comme ça que j'avais compris que, elles savaient que ça l'envoyait une notification, mais eux, ça l'avait comme message de leur bord que, en tout cas ce que j'avais retenu c'est que eux, elles devaient quand même être autonomes de nous contacter [...] nous c'était clair que, en principe, fallait attendre que la patiente nous appelle, ok, mais sauf qu'à un moment donné, vu que si la patiente ne nous appelait pas, ben là on la contactait parce que c'était quand même une toxicité qui était, qu'il y avait un certain grade."* (Entrevue 3)

Finally, participants who are not pharmacists explain that the use of the application has not had an impact on their follow-ups, the number of calls received and the duration of the follow-ups.

*"C'est difficile à dire. Probablement qu'ils ont eu un impact, mais nos patientes ne nous l'ont pas dit. Puis tu sais, je ne pense pas que mon patient ait appelé moins ou appeler plus à cause de ça. Tu sais, c'est sûr que si on avait su les noms des personnes versus les appels, peut-être qu'on aurait été capables de différencier. Mais sincèrement, je ne pense pas qu'on en a eu vraiment un impact sur les appels. Tu sais, avec toutes les lignes de conduite qu'il y avait, elles étaient bien dirigées puis je pense que ça se dirigeait plus vers les pharmaciens quand il y avait des besoins. Au niveau de la gestion des effets secondaires, c'est vraiment plus les pharmaciens présentement qui font cette gestion-là. Fait que toute, d'après moi, était dirigé vers eux* Interviewer : *"Fait que ça c'est les temps de visite ou on va dire le processus des visites. Mais est-ce que, quand vous saviez que la personne avait un rapport d'effets secondaires que vous consultiez, est-ce que vous trouviez que votre consultation ou votre interaction avec la personne était améliorée ou pas?"* Participant *: "Ça changeait pas. Il y a rien qui a changé pour moi"* (Entrevue 2)

Interviewer : *"Est-ce que vous trouvez que les rapports avaient un impact sur les temps de visite avec vos patientes? "* Participant : *"Je pense que ça n'a pas changé mon temps de visite, ce n'était pas plus long, c'était pas moins long, c'était pareil"* (Entrevue 4)

*"Non, je pense pas que, non je pense pas que ça l'a eu d'impact sur le temps des visites, ni plus long, ni plus court je dirais"* (Entrevue 1)

### 4. Implementation of the application in routine practice

All participants would like to see the application implemented in routine practice, including in the long term.

Interviewer : *"Aimeriez-vous que l'utilisation de l'application soit implantée dans la pratique courante?"* Participant : *"Oui"* Interviewer : *"J'entends que c'était une expérience positive que vous aimeriez voir continuer dans l'avenir"* Participant : *"Absolument, ben oui!"* (Entrevue 4)

### 4.1 Factors that could facilitate implementation

Participants discussed several factors that would facilitate the implementation of the application in routine practice.

One participant mentioned finding a way to identify target patients to offer the application and remind them to complete the questionnaires before their appointment with the doctor.

> *"[...] les patientes qui sont sous thérapie endocrinienne, il faudrait qu'il y ait un système pour les identifier dans les rendez-vous, puis, quelques jours avant leur rendez-vous, qu'elles reçoivent comme une alerte par courriel d'aller remplir leur questionnaire [...]"* (Entrevue 4)

One participant emphasized the importance of including the various professionals on the treatment team in discussions regarding the application's implementation. This would ensure that the needs of all professionals are taken into account and facilitate interdisciplinary collaboration in patient care.

> *"[...] avant de partir quelque chose comme ça, il faut vraiment se parler en équipe puis travailler tout le monde ensemble. Il n'y a pas juste les infirmières pivots, il y a autant les pharmaciens puis tu sais, au-delà de ça, il y a les équipes psychosociales qui sont là-dedans, qui doivent accompagner les patientes qui sont en détresse, qui sont anxieuses, ben de savoir c'est quoi, je pense que c'est important aussi pour ces personnes-là, puis l'équipe médicale, tout le monde, tu sais, c'est pas juste un silo, c'est plusieurs personnes qui doivent toutes travailler ensemble"* (Entrevue 2)

According to one participant, facilitating the application's implementation in routine practice would be easier access to adverse event reports generated by the application. Integrating these documents into the platform already used for reviewing medical records would allow for easier and more intuitive access to the summary of patient responses to the application's questionnaires.

> *Participant : "Ben c'est sûr que ce qui est facilitant, je pense, c'est que [...] quand ça entre dans la [...] trajectoire habituelle. Donc, on n'est pas obligé d'ouvrir une nouvelle fenêtre (à propos de la navigation dans le dossier médical pour consulter le rapport d'effets indésirables) [...] allez sur un autre site internet puis tout ça, fait que ça, ce qui est facilitant, c'est quand t'as un programme qui rentre dans ta trajectoire habituelle là, c'est le même dossier Cristalnet (dossier médical électronique des patients), puis ça fait juste être dans le même logiciel."* (Entrevue 1)

Providing technical support and making available a user guide with clear instructions and videos for patients and healthcare professionals are other factors that would facilitate the app's implementation, according to two other participants. This could also be achieved through mentoring or patient partners. One participant explained that in a different context, patient support from patient partners had been highly valued and could be equally appreciated by patients, women, and professionals, allowing them to better manage their visit time. This could be a good strategy to promote the explanation and implementation of the app.

> *"tu sais, autant que nous faut le comprendre puis l'expliquer aux patients, il faut les soutenir, il faut qu'il y ait une équipe de soutien parce que, tu sais, on a à répondre à nos patients, mais je pense qu'on peut pas se permettre de tout leur expliquer comment peser sur le piton puis*

*comment se connecter puis, fait que ça prend une équipe de soutien aussi. Puis plus qu'on va le faire, plus que les patients vont être habitués puis ils vont se parler entre eux, nos patientes se parlent beaucoup entre elles. Fait que ça va venir qu'à se faire tout seul, mais ça prend une équipe de soutien avant tout pour bien partir ça […] Ben tu sais, c'est sûr que présentement il y a le site internet du Centre des maladies du sein, où qu'on peut aller chercher de l'information. Tu sais, ça prendrait peut-être une petite capsule vidéo pour expliquer, si on venait qu'à utiliser ce programme-là, une petite capsules vidéo qui explique ou des screenshots qui disent comment faire. Moi je pense dans tout ça, c'est le support technique avant tout, puis de les accompagner."* (Entrevue 2)

*"Puis peut-être que ça prendrait, peut-être que pour les, oui monde on explique le projet et tout ça, je sais pas si ça prendrait quasiment […] pour aider à accompagner les patientes, il y avait des patientes qui acceptaient à faire du mentorat. Les patientes qui avaient déjà comme participé au projet ou qui avaient déjà eu telle sorte de traitement, puis que elles, c'était comme leur façon de redonner aux suivantes. Puis c'était comme, ils appelaient ça les patientes partenaires, elles étaient comme impliquées à même l'équipe pour pouvoir justement épauler les nouvelles patientes qui allaient utiliser ces applications-là, qui allaient utiliser telle nouvelle thérapie, comme un genre de groupe de soutien. Fait que ça, ils disaient que c'était super aidant. […] c'était quelque chose qui aidait beaucoup, je pense qui était apprécié là-bas.*
Interviewer*: Oui, ça serait comme un facteur facilitant pour soutenir la mise en œuvre tu sais*
Participant *: Oui exactement, oui le pharmacien, on explique nos affaires et tout ça. Mais tu sais, des fois oui, on essaie de mieux le vulgariser possible tout ça, mais là c'est aussi technique. Mais on n'est pas dans la peau de ces personnes-là justement qui ont de la détresse, etc. Fait que des fois d'avoir quelqu'un qui a porté ou qui porte encore les mêmes chaussures tu sais, finalement là, c'est comme quelque chose qui avait l'air d'avoir été apprécié"* (Entrevue 3)

Finally, one participant explained that it would be important to emphasize that using the app will not replace human contact and interactions with healthcare professionals. Informing participants that the app is a tool that allows for more effective communication of information to professionals would reassure them and alleviate their concerns.

*"[…] je pense, tu sais, faut pas qu'ils aient peur de ne plus pouvoir parler à personne, tu sais derrière un écran, derrière un téléphone, il y a tout le temps quelqu'un qui est là aussi. C'est comme un boni mais ça reste qu'il y a des individus en arrière"* (Entrevue 2)

**4.2 Perceived barriers and challenges to implementing the application in routine practice**

One participant explains that working in silos, where knowledge transfer is not carried out to either healthcare professionals or patients, is a barrier to implementing the application in routine practice.

*"Oui, ben je pense que j'ai déjà mentionné, j'avais comme l'impression que, je sais pas si la patiente s'attendait à ce qu'on en parle, mais c'est sûr que, ce que les études démontrent, c'est que les patients aiment ça savoir qu'on a regardé ce qu'ils nous ont dit. Mais là j'avais pas l'impression que les patients savaient que j'allais regarder ou que j'allais regarder, ou elles*

*semblaient pas s'attendre à ce qu'on regarde ça ensemble ou, fait que tu sais, je pense qu'il y a un gros travail de transfert des connaissances à faire, tu sais, parmi le personnel, parmi les patients aussi, éventuellement, si c'était implanté là. J'avais l'impression que c'était plus en silo actuellement là"* (Entrevue 1)

In the same vein, two participants highlighted the challenges to implementing the application in routine practice brought about by the lack of clarity and the time required to register patients, explain the application and support them in its use.

*"Ben oui c'est ça, la barrière, ça va être d'être capable d'identifier à l'avance les patientes qui sont sous thérapie endocrinienne puis de coordonner avec leurs rendez-vous, ou au moins de faire une banque de patientes sous thérapie endocrinienne, puis après ça, avoir leurs coordonnées pour leur envoyer le questionnaire régulièrement, de façon régulière, puis qu'après ça, nous comme médecin, on le reçoive où que ça soit disponible dans le dossier assez rapidement, parce que si la patiente remplit son questionnaire mais que ça arrive trois mois plus tard dans le dossier, on n'est pas plus avancé. Fait que ça, c'est un autre enjeu qui peut être"* Interviewer*: "Idéalement, ça serait quoi le laps de temps pour que ça rentre dans le dossier? Participant : "Ben il faudrait que ce soit 24h"* (Entrevue 4)

*"Puis il y a aussi d'expliquer aux patientes comment ça va fonctionner. Fait que ça, c'est sûr que c'est du temps aussi, fait que ça c'est un peu une barrière je dirais. Si quelqu'un leur expliquait puis qu'elles pouvaient s'inscrire toutes seules, puis qu'on aurait juste à gérer les alertes, moi je verrais ça très, ben facile, oui"* (Entrevue 5)

*"Ben des barrières, c'est que, malheureusement, c'est peut-être pas, ben moi comme professionnelle, c'est moi qui faisais le recrutement, qui inscrivais les patientes han, fait que ça c'était peu fastidieux ce côté-là, fait que ça, c'est certain que ça c'est pas facile. Ça me demande beaucoup de temps d'inscrire, après ça, mais c'est aussi parce que c'était dans le cadre d'une étude de, après ça, d'attendre d'avoir le formulaire de consentement signé pour pouvoir ensuite envoyer les questionnaires. Fait que c'est sûr que dans la pratique courante, ben il y aurait probablement pas de formulaire de consentement, ça serait peut-être plus facile, mais quand même, juste cette étape-là d'inscrire tout le monde là-dessus, je trouve que c'est un peu une barrière. Moi, si les patientes pouvaient s'inscrire elles-mêmes à quelque chose comme ça, ça serait plus, ça serait facilitant. Fait que ça de mon côté, pour disons partir l'affaire, ça c'est quand même un peu complexe. "* (Entrevue 5)

*"Les barrières je pense, c'est surtout au point de vue technique pour les inscriptions pour les patientes, je pense que c'était surtout ça, des fois qu'il y en a qui rappelaient dans la première semaine là, elles disaient – Ah là, comment ça fonctionne là, je suis pas sûre, en tout cas, c'est un peu ça que j'ai un peu entendu, mais sinon il y avait pas vraiment de messages de problèmes, j'avais pas entendu d'autres problèmes en tant que tels. Il n'y a pas de patientes qui ont dit que c'était trop lourd, rien du tout là de ce côté-là"* (Entrevue 3)

According to one participant, patients who will find it more difficult to integrate the application into their clinical care routine will be older patients, those with low technological literacy, or those with cognitive impairments. Strategies to facilitate access to the application, such as making a tablet available in waiting rooms or providing support from a caregiver, were suggested.

> *"[...] Puis les barrières, ben ce sera toujours les barrières informatiques puis tout ça."* (Entrevue 1)

> *"[...] ça pourrait être d'avoir des IPad dans la salle d'attente qui seraient disponibles justement puis ce serait écrit – N'oubliez pas de remplir votre questionnaire avant votre visite avec le médecin pour celles qui prennent tel médicament. Tu sais, il faut que l'outil soit accessible facilement aux patientes, puis pour celles qui n'ont pas accès mettons à un ordinateur ou qui ne sont pas habiles avec justement la technologie, ben peut-être que la formule IPad dans la salle d'attente est plus facile d'accès que, elles, aillent sur leur, tu sais, peut-être qu'elles en n'ont même pas de téléphone intelligent ou de tablette ou quoi que ce soit, fait que des fois ça peut être plus difficile de ce côté-là. D'un autre côté, pour celles qui sont vraiment habiles avec les outils, ben juste d'avoir un lien courriel, après ça, elles peuvent l'ouvrir n'importe quand elles ont le temps, puis elles remplissent ça"* (Entrevue 4)

> *"Eh, peut-être que si c'était le proche aidant qui pouvait peut-être, ou leurs enfants qui peuvent aider à les suivre à distance, sans nécessairement être un suivi aussi strict que d'avoir à répondre à certains questionnaires à tous les jours ou une fois par semaine. Mais si leurs enfants voudraient l'utiliser, par exemple quand ils les appellent, ils pourraient mettre les informations eux autres dans l'application. Ça ferait sûrement quelque chose qui serait faisable si la personne, la patiente en tant que telle, elle était moins d'adon, qu'elle avait moins les outils technologiques. Je pense que ça pourrait être une idée là."* (Entrevue 3)

> *"[...] vraiment la grosse barrière, c'est la barrière informatique là. Ben il y a deux barrières, 1) c'est qu'on n'a pas d'outil vraiment, le bon outil électronique, on a utilisé Redcap, mais ça faisait, mais c'est pas l'idéal, on n'a pas de graphique, c'est pas en temps réel. Bon, c'est un peu compliqué. Puis aussi, on avait sous-estimé aussi une assez bonne proportion de patientes qui n'ont pas participé, parce qu'elles n'étaient pas, elles se sentaient pas assez à l'aise avec l'informatique. Fait que ça, ça été plus élevé qu'on pensait là. Mais oui, c'est sûr qu'à terme, je pense que ça serait très intéressant d'avoir ça en pratique si on avait un bel outil informatique là"* (Entrevue 1)

> *"Ben tu sais, je pense que les personnes âgées, c'est un gros enjeu parce qu'ils ne sont pas encore habitués avec la technologie, quoi qu'il y a des jeunes aussi qui ont de la misère. Tu sais, c'est pas donné à tout le monde. La barrière, je pense, c'est la technologie. Ça peut faire peur à quelqu'un de pitonner ça. Je pense que ça peut être vraiment un des premiers enjeux dans tout ça. "* (Entrevue 2)

Another challenge to the implementation of the application, according to two participants, would be the difficulty in identifying patients who use the application and whose adverse event reports are available.

> *"la barrière, ça va être d'être capable d'identifier à l'avance les patientes qui sont sous thérapie endocrinienne puis de coordonner avec leurs rendez-vous, ou au moins de faire une banque de patientes sous thérapie endocrinienne, puis après ça, avoir leurs coordonnées pour leur envoyer le questionnaire régulièrement, de façon régulière, puis qu'après ça, nous comme médecin, on le reçoive où que ça soit disponible dans le dossier assez rapidement, parce que si la patiente remplit son questionnaire mais que ça arrive trois mois plus tard dans le dossier, on n'est pas plus avancé. Fait que ça, c'est un autre enjeu qui peut être* (Entrevue 4)

> *"[...] Puis il faut savoir aussi que la patiente participe, des fois, c'est pas toujours évident là, si ça devenait routinier, ben on le saurait, mais là actuellement c'était pas, mettons que c'était pas évident là. Moi je le savais parce que c'était mon projet fait que j'étais vraiment impliquée là. Mais pour quelqu'un qui a juste mis une ou deux patientes, ça devait pas être évident de savoir si la patiente participait ou non. Puis si tu voyais la patiente de quelqu'un d'autre, ben c'était pas évident non plus là. Fait que je trouvais que, c'est ça, les patientes l'abordaient pas spontanément non plus. Moi mon expérience c'est que les patientes spontanément me disaient pas – Ah j'ai utilisé l'application, il fallait plus que je pose des questions aux patientes"* (Entrevue 1)

> *" [...] j'avais comme l'impression que, je sais pas si la patiente s'attendait à ce qu'on en parle, mais c'est sûr que, ce que les études démontrent, c'est que les patients aiment ça savoir qu'on a regardé ce qu'ils nous ont dit. Mais là j'avais pas l'impression que les patients savaient que j'allais regarder ou que j'allais regarder, ou elles semblaient pas s'attendre à ce qu'on regarde ça ensemble"* (Entrevue 1)

**5. Added Value of the Application**

According to one participant, the added value of using the application in routine practice is to limit less relevant interactions with healthcare professionals. More specifically, this would allow patients to become more autonomous in managing their side effects.

> *"pour nous, ben tu sais, les patients vont le manipuler puis le connaître plus, ça fait qu'ils vont avoir des lignes de conduite au lieu de nous contacter, puis c'est des affaires qu'ils peuvent mettre en pratique par eux-mêmes. Fait que moi je pense que oui, ça serait quelque chose de vraiment pratique. On le voit, ça se fait dans d'autres centres hospitaliers puis c'est quand même un bon, c'est positif [...]* (Entrevue 2)

For another participant, the application would be an effective tool to facilitate the screening and assessment of adverse effects in routine practice.

> *"Mais je dirais, ça va faciliter ton travail parce que le patient va déjà avoir pensé aux toxicités qu'il a. Il va les avoir gradées, puis toi après ça, ben t'as juste à adresser les effets secondaires que le patient a, au lieu d'essayer de faire, d'aller à la pêche avec ton questionnaire puis dire –*

*Avez-vous eu, avez-vous des antalgies? Avez-vous des bouffées de chaleur? Avez-vous ci? Là, le patient a déjà fait ce travail-là puis il t'arrive – Ben moi c'est ça que j'ai puis c'est ça qui me dérange le plus. Fait qu'aujourd'hui ben on va l'adresser ensemble"* (Entrevue 4)

One participant explains that the application helps reassure patients about their prompt care and the monitoring of side effects.

> *"Ben oui, c'est sûr que ça serait quelque chose (à propos de la disponibilité de l'application en pratique courante) quand même de rassurant auprès des patients, parce que tu sais, ils savent que la fin de semaine, on n'est pas là, ça fait que tu sais, en faisant cette ligne de conduite-là, ils sont capables d'avoir une réponse plus rapidement que de nous attendre le lundi matin ou. Fait que oui, je pense que ça serait rassurant pour les patients […]"* (Entrevue 2)

According to two participants, the application's format and tools, such as email alerts, allow for easier and more proactive management of patient concerns/follow-up compared to managing calls and paper documents usually in practice.

> *"[…] (à propos de l'utilisation de l'application en pratique courante) c'est quand même assez facile à gérer parce que, ce qu'on recevait, c'est un courriel d'alerte de l'appli. Puis en temps normal, ce qu'on reçoit c'est soit rien, parce que les patientes oublient de nous appeler ou ils n'ont pas d'effets secondaires, ou c'est un téléphone. Fait que je trouvais qu'un courriel par rapport à un téléphone, c'est plus facile à gérer je pense. Moi j'aime mieux gérer des courriels que des téléphones"* (Entrevue 5)
>
> *"[…] de plus en plus de gens sont rendus familiers avec ça (les outils technologiques sur plateformes électroniques), puis les papiers, là c'est problématique aussi la gestion à distance que là, les outils papier, on les envoyait par courriel, fait que là les gens ont pas nécessairement d'imprimante, etc. Fait que là, même pour tout le monde, on est tous plus rendus familiers avec les utilisations sur les tablettes et autres là. Fait que donc, je pense qu'on est rendus là. Puis je sais qu'il y a quand même différents projets aussi qui se font ailleurs au Québec en ce sens-là, pour justement la gestion des suivis des effets indésirables de façon proactive là"* (Entrevue 3)

**6. Recommendation of the application to colleagues**

Participants would recommend the application to their colleagues. One participant expressed some reservations about its current form. He explained that if adverse event reports were more easily and quickly accessible (via the electronic patient record used in routine practice), he would have no hesitation in recommending it. Another participant emphasized that using the application would not replace in-person follow-ups with patients, but rather be a valuable complement to facilitate the management of patient adverse events.

> Interviewer *: "Est-ce que vous présentement, vous recommanderiez l'utilisation de l'application pour un suivi des effets secondaires du traitement contre le cancer en comprimés à d'autres collègues?"*
> Participant *: "Ben oui, absolument"* (Entrevue 4)

Interviewer*: "Ça c'est bien, puis recommanderiez-vous l'utilisation de l'application en suivi des effets secondaires pour les traitements de cancer à d'autres collègues? Est-ce que c'est quelque chose que vous recommanderiez? "*Participant *: "Je dirais pas dans sa forme actuelle, parce que dans sa forme actuelle, je reviens toujours au problème informatique, ça marche pas. Tu peux pas avoir un rapport qui est rentré deux semaines plus tard, puis une page qui rentre dans le résumé extérieur, qui est pas dans l'onglet Clinique du sein, ça marche pas là. Fait que je vous dirais que dans la forme actuelle, non, mais tous les algorithmes qui ont été développés, ça c'est super, c'est juste qu'il faudrait qu'ils soient dans un autre outil informatique, mais je dirais pas dans la forme actuelle, point de vue de l'image, puis tout ça, mais oui, certainement, je dirais le même contenu, mais dans un autre contenant là"* (Entrevue 1)

Interviewer*: "Puis est-ce que vous recommanderiez l'utilisation de l'appli en suivi des effets secondaires pour des traitements contre le cancer avec des comprimés à d'autres de vos collègues? "*
Participant : *"Eh oui, sans problème, oui, tout à fait. Oui, c'est sûr que toutes celles ou ceux qui étaient éligibles avec des nouveaux traitements, ben on leur proposait là, c'est certain. C'est sûr que ça l'empêche pas, tu sais, ça annule pas le suivi après ça qu'ils doivent nous contacter, ça c'est certain. Mais oui, d'avoir certains outils, certains rappels de gestion d'effets indésirables, un peu comme des conseils de base qui vont être inclus dans le système, c'est sûr que ça permet d'être facilitant, oui"* (Entrevue 3)

**7. Suggestions for Improving the App**

Several suggestions were offered by participants to improve the app.

One participant suggested adding a medication tracking tool to the app in the form of an alert to facilitate adherence to treatment, particularly when there are different phases in medication intake.

Interviewer*: "Puis avez-vous d'autres idées qui pourraient être faites, mises en place pour améliorer l'appli telle que vous l'avez expérimentée? "* Participant *: "Mon dieu. Peut-être un outil d'adhésion au traitement que ça pourrait être intéressé, je sais que Sophie est assez, elle le sait, un de ses dada de ce côté-là. S'il y avait un outil de prise de la médication, comme un système de calendrier, tu sais pas juste le volet gestion effets désirables, mais aussi un volet adhésion au traitement, fait que ça serait un outil, soit avec des possibilités de se mettre une alarme sur le téléphone pour prendre sa médication, avec le moment de la prise, surtout quand il y a des traitements qui sont séquentiels. Puis de plus en plus, on s'en va vers ça, tu sais. Ben pas un traitement en continu une fois par jour le matin, ça c'est simple, puis ça, habituellement ça va bien, mais c'est quand justement, il y a des séquences de traitements, deux semaines sur trois, trois semaines sur quatre, là maintenant, il y en a un nouveau traitement que j'ai entendu la semaine passée, ça va être quatre jours, suivis de trois journées de congé, trois semaines sur quatre, là c'est du lundi au jeudi, fait que faut pas se tromper dans le calendrier là han [...] Parce que je pense que ça serait quelque chose qui serait intéressant. Puis, si la personne, elle a pas fait le crochet qu'elle l'a pris, ben là il y a peut-être comme un rappel qui est fait et qui*

*dit – Oh, avez-vous pris votre médicament ce matin? – Oh non c'est vrai, j'ai oublié. Fait que là, ça serait comme un outil qui serait intéressant"* (Entrevue 3)

One participant suggested expanding the assessment to include side effects of the disease in addition to adverse effects of treatments. They explained that disease progression plays a role in medication adjustment decisions and that being able to assess all symptoms beyond medication side effects could promote more comprehensive medication management. Similarly, another participant suggested integrating a section into the application for accessing the patient's test results, as well as expanding the application's use to treatments other than oral chemotherapy, as mentioned by two participants. One of these participants explained that introducing the application early in the care pathway, for example, with intravenous chemotherapy, would make women feel more comfortable using it at home for other treatments. However, they felt that introducing the application during the first cycle of oral treatment might be too burdensome for patients who already have too many new things to manage simultaneously. Introducing it during the second treatment cycle would be a more suitable option.

> Interviewer*: "Puis avez-vous d'autres idées qui pourraient être faites pour mieux accompagner ou outiller les femmes qui reçoivent une prescription d'un traitement contre le cancer en comprimés suite à un cancer du sein? Des choses dans l'appli qui peuvent être améliorées ou d'autres choses que l'appli qui pourraient être mis autour? "* Participant *: "Ben c'est certain que l'appli, on se concentre, on voulait pas refaire un projet qui avait déjà été fait. Puis parmi les projets qui avaient déjà été faits, il y avait des projets que, où est-ce qu'il y a tous les symptômes, pas juste les effets indésirables de la médication, comme par exemple la fatigue, d'autres choses qui peuvent, l'essoufflement, qui sont pas nécessairement en lien avec les, qui ne sont pas des effets secondaires nécessairement du traitement, mais souvent des effets secondaires ou, en tout cas, qui peuvent être des effets secondaires de la maladie. Fait que ça, je pense que ça serait peut-être bien d'ajouter ça, ce genre de question-là aussi, parce que même si c'est pas un effet secondaire du médicament, des fois ça va nous amener à faire des modifications de doses, des pauses, etc., devancer un rendez-vous médical. Fait que ça c'est certain que ça serait un ajout qui serait probablement souhaitable. Et puis s'il y a un ajout comme ça toutefois, ben là, je pense qu'on serait peut-être pu capable de le gérer juste la pharmacie, ça s'est fait, ça été géré par la pharmacie parce que c'était des effets secondaires spécifiques à des molécules qu'on est habitués de gérer au téléphone. Mais si ça s'étendait à d'autres symptômes, là je pense qu'il faudrait impliquer d'autres professionnels, fait que, il y aurait ça que je vois, mais en même temps, il y a certaines limites aussi. Fait que c'est positif, mais ça demande beaucoup de temps tout ça. C'est ça, de l'expliquer aux patientes, d'inscrire les patientes, puis ensuite de regarder les réponses des patientes"* (Entrevue 5)

> *"puis c'est des affaires qu'ils peuvent mettre en pratique par eux-mêmes. Fait que moi je pense que oui, ça serait quelque chose de vraiment pratique. On le voit, ça se fait dans d'autres centres hospitaliers puis c'est quand même un bon, c'est positif, puis peut-être éventuellement de le bonifier, tu sais d'avoir une application bonifiée ou qui pourrait avoir leur formule sanguine dedans et tout. Moi je pense que ça serait une belle plateforme pour nos patients [...] Ben tu sais, c'est un super beau projet, on n'est pas encore habitué à ce genre de travail-là de logiciel, ça fait que c'est de travailler avec, c'est de le peaufiner. Tu sais, il y a jamais rien de*

*parfait, fait que de l'upgrader, de le tenir à jour puis de rajouter des choses, tu sais, avec les patients, c'est plus qu'on va le travailler plus que les patients vont nous dire quoi. Là on avait développé certaines conditions, certains effets secondaires, mais tu sais, c'est sûr que ça pourrait être encore plus large. Puis au-delà de la thérapie de la chimio per os, on pourrait même aller développer les hormonothérapies où qu'on a beaucoup d'effets secondaires avec la gestion de l'hormonothérapie qui est très, quand même difficile à prendre pour les patientes. Puis ça, je pense que ça serait le prochain volet à développer pour bien les outiller. Fait que tu sais, il pourrait être encore plus développé, plus grand puis apporter plus d'informations. Mais tu sais, c'est un travail de, c'est un gros travail. Je sais que la pharmacienne a travaillé beaucoup sur tout ça, c'est vraiment quelque chose de gros, mais je pense qu'on peut avoir un plus dans tout ça au fil des années"* (Entrevue 2)

*"Je pense que non. Pourtant, j'ai été impliquée beaucoup han. Non, vraiment t'as posé des questions puis j'ai vraiment dit ce que j'avais là, l'espèce de lourdeur, tout ça, mais peut-être un questionnement que j'ai c'est que, on a fait des algorithmes pour beaucoup de médicaments, est-ce que, ben je suis un petit peu curieuse de savoir est-ce que c'est vraiment pour tous les médicaments que ça devrait ou on devrait peut-être cibler certains médicaments, fait que ça c'est un point d'interrogation que j'ai. Peut-être qu'au contraire, ça serait bon pour tous les médicaments, puis encore plus de symptômes. Je sais pas si on pourrait retirer de meilleurs bénéfices en ciblant plus ou en étendant plus. "* (Entrevue 5)

Interviewer*: "Puis il y a tu d'autres éléments que vous aimeriez apporter à la connaissance de l'équipe de recherche concernant votre expérience d'utilisation de l'appli avec vos patientes? "*
Participant *: "J'avoue moi je l'ai pas manipulée l'application moi-même évidemment. Mais c'est que, on se rendait compte que, je trouvais qu'il y avait beaucoup de patientes qui ont, je pense, qui ont décidé de pas utiliser l'application. Elles disaient – Ah là ça fait déjà trop pour moi là, déjà là, oui nouveau traitement puis souvent, c'était, mettons un exemple, une première ligne de cancer du sein métastatique. Là, ça faisait quand même beaucoup de choses, un peu un choc, etc., fait que là, elles trouvaient que ça faisait un peu trop tu sais. Mais bon, fait que peut être si, là mettons, on aurait un deuxième traitement, si exemple il y a une progression, puis là on leur offre un deuxième traitement, là peut-être qu'elles auraient été peut-être plus enclines, mais peut-être que certaines patientes, là, ça l'a moins embarqué à cause de ça. Ça faisait peut-être un peu trop là pour commencer. Mais bon, fait que ça, on verra. Peut-être que si justement, l'application, là oui, c'était pour les traitements en pilules, mais si ce genre d'application-là était aussi, créée exemple pour des traitements intraveineux en première ligne par exemple, puis qui étaient déjà familières avec ces outils-là, fait que ça serait un peu comme une continuité de, fait que ça serait de l'intégrer je pense même plus large que le traitement en pilules, ça serait comme un, déjà faut commencer à quelque part han évidemment là. Mais là, c'est sûr que c'est un peu plus large puis des fois moins ciblées, mais ça serait comme l'autre volet qui permettrait d'encore mieux intégrer, qui serait disponible pour finalement toutes, peu importe le type de cancer, en pilules puis intraveineux"* (Entrevue 3)

Finally, one participant suggested improving the layout of adverse event reports to make them easier and more intuitive to read. This would be achieved through color coding and graphs showing changes

over time. This would also allow the document to be shared with the patient, who could then interpret it more easily.

> *"Non, c'était quand même facile à lire, c'était assez aéré là. C'est juste que c'était pas des rapports, par exemple, il n'y avait pas de graphique. Ça aurait été intéressant de voir des graphiques qui comparent d'une semaine à l'autre l'évolution des symptômes. On aurait pu avoir des codes couleurs puis tout ça, mais bon, malheureusement c'était juste la question avec le score qui était écrit en lettres ou en chiffres donc, mais bon, ça on le savait quand on a commencé le projet. Mais tu sais, à terme, c'est sûr qu'on voudrait avoir quelque chose qui est mieux visuellement puis qu'on peut partager avec les patientes aussi. Parce que là, on partageait pas nécessairement le rapport avec la patiente, moi je regardais son rapport, disons je voyais – Avez-vous de la diarrhée? Mettons elle mettait grade 2, mais je montrais pas l'écran pour lui montrer. Peut-être que si on avait eu des graphiques avec l'évolution au cours des semaines, ben peut-être qu'on aurait pu le regarder ensemble, mais là ça se prêtait pas vraiment là"* (Entrevue 1)

**8. Suggestions for improving adverse event monitoring – beyond the application**

One participant suggested implementing a specialized adverse event management clinic, modeled after one already established in Ontario. This would reduce the workload of oncologists while ensuring specific monitoring of adverse events and continuity of care.

> *"D'autres idées. Ben je reviens tout le temps avec ça mais, quand j'ai fait mon fellow à Toronto, il y avait une clinique de survivance puis ces patientes-là étaient envoyées à la clinique spéciale, la clinique de survivance du cancer, qui était gérée par des médecins de famille spécialisés en oncologie. Puis c'était vraiment, les patientes étaient suivies et vues pour justement la gestion des toxicités reliées à ces traitements-là endocriniens adjuvants. 1) ça décharge le travail de l'oncologue qui, tu sais nous des fois, pas qu'on minimise, mais dans le sens qu'on a tellement la tête prise dans les patients qui sont sous chimiothérapie puis les nouveaux protocoles que les patientes après ça qu'on suit après, que ça fait 2-3 ans qu'elles sont sous inhibiteur de l'aromatase puis là, elles ont des effets secondaires, des fois on est comme, on prend moins le temps pour ces patientes-là alors qu'elles en ont tout autant besoin. Fait que moi je trouvais ce qui était vraiment cool, c'est que ces patientes-là, à Toronto, on les référait à la clinique de survivance, puis c'était vraiment des médecins qui étaient spécialisés là-dedans, de famille, qui prenaient le temps de s'asseoir avec les patientes, puis d'adresser tous leurs enjeux via une clinique de survivance. Puis je trouvais que c'était vraiment efficace. 1) ça permettait aux oncologues de faire leur travail, de traiter les patientes avec de la chimio, mais ça faisait pas en sorte que ces patientes-là étaient perdues non plus dans la nature"* (Entrevue 4)
